# *Escherichia coli* Can Eat DNA as an Excellent Nitrogen Source to Grow Quickly

**DOI:** 10.3389/fmicb.2022.894849

**Published:** 2022-06-28

**Authors:** Lili Huang, Yehui Zhang, Xinmei Du, Ran An, Xingguo Liang

**Affiliations:** ^1^College of Food Science and Engineering, Ocean University of China, Qingdao, China; ^2^Laboratory for Marine Drugs and Bioproducts, Qingdao National Laboratory for Marine Science and Technology, Qingdao, China

**Keywords:** DNA, *Escherichia coli*, nitrogen source, nutrient, nutrition

## Abstract

Is DNA or RNA a good nutrient? Although scientists have raised this question for dozens of years, few textbooks mention the nutritional role of nucleic acids. Paradoxically, mononucleotides are widely added to infant formula milk and animal feed. Interestingly, competent bacteria can bind and ingest extracellular DNA and even integrate it into their genome. These results prompt us to clarify whether bacteria can “eat” DNA as food. We found that *Escherichia coli* can grow well in the medium with DNA as carbon and nitrogen sources. More interestingly, in the presence of glucose and DNA, bacteria grew more rapidly, showing that bacteria can use DNA as an excellent nitrogen source. Surprisingly, the amount of DNA in the culture media decreased but its length remained unchanged, demonstrating that *E. coli* ingested long DNA directly. The gene expression study shows that *E. coli* mainly ingests DNA before digestion and digests it in the periplasm. *Bifidobacterium bifidum* can also use DNA as the nitrogen source for growth, but not efficiently as *E. coli*. This study is of great significance to study DNA metabolism and utilization in organisms. It also lays a foundation to understand the nutritional function of DNA in intestinal flora and human health.

## Introduction

In the natural environment, extracellular or free DNA can be found everywhere, but its utilization by organisms has not been clarified. In the soil, for example, DNA concentration is between 0.08 and 2 μg/g (Nielsen et al., 2007). In the ocean, 0.044 mg/L of DNA has been found (Lorenz and Wackernagel, 1994). Extracellular DNA is circulating throughout animal bodies in the blood and other fluids, including urine, milk, amniotic fluid, and bronchial lavage (Hoskins, 1978; Vlassov et al., 2007). It is also present in the wound contaminated by bacteria (Brandt et al., 1995; Ulmer et al., 1996). In contrast, bacteria are also everywhere and can utilize almost any organic molecules from organisms as “food.” However, few textbooks on nutrition mention the nutritional role of nucleic acids, although scientists have raised the question of “Is DNA or RNA a good nutrient?” for dozens of years. Paradoxically, mononucleotides are added to infant formula milk as the essential component, and RNA-rich formula is widely used in the feed of animal offspring. DNA and RNA products have also been used as functional foods. One reason for nucleic acids being ignored as nutrition is that researchers believe its content (especially for DNA) is less than proteins or polysaccharides. Actually, it is a misunderstanding, especially in the world of prokaryotes. In an *Escherichia coli* cell, for example, nucleic acids account for about 7% of the wet weight, and it is only less than protein, which is 15% (Lewin and Dover, 2002). The content of DNA in *E. coli* is about 1.0% (0.5–1.5% wet weight). From the perspective of chemical structure, it is easy to see that nucleic acid and its derivatives are higher in carbon (C), nitrogen (N), and phosphate (P). Sulfur (S) has also been found in some bacteria. For example, Deng et al. have found that the genome of many bacteria involves sulfur atoms attaching to phosphates on DNA (Zhou et al., 2005). Accordingly, it is worth studying nucleic acids deeply from the perspective of nutrition, not just from that of molecular biology and biotechnology. Many exciting findings can be expected in the areas of nucleic acid metabolism and nutrition.

Bacteria have been shown to utilize DNA as element sources of P, C, and N for growth under special conditions (Paul et al., 1988; Jørgensen et al., 1993; Redfield, 1993; Kroer et al., 1994; Lennon, 2007; Pinchuk et al., 2008; Mulcahy et al., 2010; Seper et al., 2011; McDonough et al., 2016). For example, *V. cholera* has been shown to assimilate phosphate from extracellular DNA and nucleotides, and PhoX, UshA, and CpdB have been identified as the major periplasmic phosphatases (McDonough et al., 2016). The extracellular deoxyribonucleases of Dns and Xds digest DNA first before assimilation (Seper et al., 2011). The genus Shewanella, one kind of metal-reducing bacteria, could use extracellular DNA as carbon and energy sources (Pinchuk et al., 2008). The possibility of digesting DNA into nucleotides for direct DNA and RNA synthesis was also discussed (Paul et al., 1988; Jørgensen et al., 1993; Redfield, 1993; Kroer et al., 1994). Mulcahy et al. (2010) showed that *P. aeruginosa* may secrete an extracellular deoxyribonuclease (DNase) to degrade extracellular DNA and utilize it as the C, N, and P source. Some marine bacteria were also reported to utilize DNA as a nutrient source of C, P, and N, but the culture media contained HEPES or other compounds of organic carbon and nitrogen (Lennon, 2007). *Escherichia coli*, one model bacterium for molecular biology, has also been shown to be capable of consuming DNA as the sole source of carbon and energy (Palchevskiy and Finkel, 2006). However, these studies on DNA utilization are scattered and not so persuasive from the viewpoint of nutrition. We did not find any research report using DNA as the nitrogen source in the presence of other carbon sources, although the content of nitrogen in DNA is even higher than some amino acids. It is well known that glucose is the best carbon source for most organisms, and DNA cannot be a better carbon source than glucose. Actually, there are a large number of plants in the natural environment that provide potential carbon sources, but the nitrogen source is relatively poor.

From the viewpoint of genomics, bacteria were believed to “eat” extracellular dsDNA for recombination (or for “sex”) (Goodgal, 1982; Kahn and Smith, 1984; Dubnau, 1991, 1999; Redfield et al., 1997). Later, Finkel et al. showed that the mechanisms of DNA uptake may have been widely conserved during evolution (Finkel and Kolter, 2001; Palchevskiy and Finkel, 2006). Very interestingly, competent bacteria were found to bind and transport long extracellular DNA through their cell envelope and integrate the “eaten” DNA into their chromosome (Smith et al., 1981; Solomon and Grossman, 1996; Palchevskiy and Finkel, 2006). The molecular machines set on the cell surface work for this transportation using the energy from ATP (Finkel and Kolter, 2001). More interestingly, a model was proposed that the two DNA strands are separated in the periplasm, and one strand breaks down there and the other ssDNA strand transfers to the cytoplasm. The phosphatase can remove phosphate groups from 5′- or 3′-nucleotides in the periplasm (Roy et al., 1982; Rittmann et al., 2005; Pinchuk et al., 2008; McDonough et al., 2016). Released phosphate can traverse the inner membrane *via* the phosphate-specific transport system (Pst/PhoU) (McDonough et al., 2014), and nucleosides can pass through nucleoside transporters (e.g., NupC). There are controversies about the biological significance (for evolution, repair, or nutrition) of this efficient and active intake of dsDNA (Seitz and Blokesch, 2013).

In theory, as the most significant biomolecule, DNA should be an excellent nutrient. However, there is no strong evidence, especially considering that the amount of DNA is usually trivial in a cell. To solve this conflict, a breakthrough in understanding DNA nutrition is highly expected. In this study, we found DNA can be utilized as an excellent nitrogen source by *E. coli*, one of the most important model bacteria. When cultured in the coexistence of glucose and DNA (as the sole nitrogen source), *E. coli* grew unexpectedly quickly. As the nitrogen source, DNA was even comparable to glutamic acid, an amino acid. We also found that DNA was “eaten” directly and efficiently without degradation. We believe that the ability of *E. coli* to assimilate DNA as a nutrient indicates that bacteria utilize DNA very actively as a “delicious” food ingredient of high quality. A model for how *E. coli* assimilates and utilizes DNA is proposed.

## Materials and Methods

### Materials

Salmon sperm DNA (GC content is 41.20%, 100–250 bp) was purchased from Tokyo Pharmaceutical Factory Limited (Tokyo, Japan) and was solved in water to a concentration of 10.0 g/L. The purity of nucleic acid was evaluated using UV-Vis spectra. At 280 nm, no absorption peak was observed, and the Abs_260_/Abs_280_ was higher than 1.8 (Supplementary Figure 1). Oligo DNA nutrients for culturing DNA or as primers for RT-PCR used in this study (see sequences in Supplementary Table 1) were synthesized by Sangon Biotech Co., Ltd. (Shanghai, China). *Staphylococcus aureus* (ATCC6538) was purchased from the China Center of Industrial Culture Collection (CICC). *Escherichia coli* (No. 1.3344) and *Bifidobacterium bifidum* (*B. bifidum*, No. 1.5091) were purchased from the China General Microbiological Culture Collection Center (CGMCC). Media (nutrient broth and TPY broth) were purchased from Hope Bio-technology (Qingdao, China). Nutrient broth (pH 7.0 ± 0.2) contains the following components (per liter): peptone 10.0 g, beef extract powder 3.0 g, NaCl 5.0 g; TPY broth (pH 6.5 ± 0.1) contains (per liter) casein hydrolysate 10.0 g, peptone from soybean meal 5.0 g, yeast extract 2.0 g, glutose 5.0 g, L-cysteine monohydrochloride 0.5 g, K_2_HPO_4_ 2.0 g, MgCl_2_ 0.5 g, ZnSO_4_ 0.25 g, CaCl_2_ 0.15 g, FeCl_3_ 0.10 mg, and Tween-80 1.0 ml. Glutamic acid (98.5%) was purchased from Sinopharm Co., Ltd. (Beijing, China). For all plates, agar was added to a concentration of 15.0 g/L. DNase was purchased from Tiandz Gene Technology Co., Ltd. (Beijing, China). Proteinase K (20.0 g/L) was purchased from Sangon Biotech Co., Ltd. (Shanghai, China). Other chemical reagents such as HClO_4_, phenol/chloroform/isoamyl alcohol, were purchased from Solarbio Science & Technology Co., Ltd. (Beijing, China). The RNA prep pure kit and FastKing RT kit for RNA extraction and reverse transcription were purchased from Tiangen Biotech Co., Ltd. (Beijing, China).

### Culture of Bacterial Strains in Various Media

First, *E. coli* were cultured in nutrient broth at 37°C under atmospheric conditions, whereas *B. bifidum* was cultured in TPY broth at 37°C under anaerobic conditions in an anaerobic incubator (Mitsubishi Gas Chemical, Tokyo, Japan). To ensure that no other residual sources of carbon and nitrogen remained in the cultures, bacterial strains were then propagated in an M9 minimal medium with glucose as the source of carbon and NH_4_Cl as the source of nitrogen. This defined growth medium (pH 7.0 ± 0.2) contains 22.0 mM glucose (4.0 g/L), 18.6 mM NH_4_Cl, 22.0 mM KH_2_PO_4_, 0.10 mM CaCl_2_, 47.0 mM Na_2_HPO_4_, 8.5 mM NaCl, and 2.0 mM MgSO_4_.

For nucleic acid-dependent growth using various nucleic acid derivatives (including DNA, deoxyribonucleotides (dNMPs), and deoxyribonucleosides) as the sources of carbon and nitrogen, nucleic acid was added to the carbon or/and nitrogen-free medium to a final concentration ranging from 0.10 to 10.0 g/L. The nucleic acid was added to the medium prior to filtering through a 0.22-μm-pore-size membrane (Jinteng, Tianjin, China). The proteinase K was used to remove any potentially contaminated protein present in the commercial DNA preparation under the following conditions (10.0 ml): 10.0 g/L DNA, 0.5 g/L of proteinase K, 37°C, 30 min, 10.0 mM NaH_2_PO_4_ (pH 7.5), 5.0 mM CaCl_2_. After the reaction, the solution was extracted by phenol/chloroform/isoamyl alcohol and chloroform/isoamyl alcohol, followed by ethanol precipitation.

### Growth Curves of *E. coli* With DNA, Deoxyribonucleotides, and Deoxyribonucleosides

Initially, the overnight cultures were taken out of the incubator and put on ice to block the growth of bacteria. After cooling on ice for 15 min, the colonized bacteria strains were centrifuged (4,500 g, 4°C, 10 min) and washed three times with 10.0 ml of the corresponding basal medium (without DNA, dNMPs, or deoxyribonucleosides). Then the cells were harvested, and resuspended in the basal medium, followed by the addition of the nucleic acid. All bacterial strains were adjusted by the medium to an optical density (OD) of 0.05 (~1.0 × 10^8^ CFU/ml) at 600 nm, and then incubated aerobically (for *E. coli*) or anaerobically (for *B. bifidum*) at 37°C. The number of *E. coli* (CFU/ml) values were calculated using a standard curve obtained by counting the colonies on an agar plate. For analyzing samples cultured for various time intervals, each of the bacteria suspensions (200 μl) was taken and added to a 96-well plate and measured the OD at 600 nm and 37°C immediately (in some cases, if the OD value exceeds 0.8, the samples are further diluted to the OD value between 0.1 and 0.8). All growth experiments were independently repeated at least three times. The error for OD values is within 4–18%. Cultures were grown in nutrient broth, TPY broth, or M9 minimal medium lacking DNA, dNMPs, or deoxyribonucleosides served as the control.

### Decrease in Absorbance at 260 nm of Exogenous Nucleic Acid During Culture

Cultures were grown in nutrient broth, TPY broth, and corresponding basal medium (containing nucleic acid) at 37°C for the same cultivation time intervals. The cell-free supernatants (CFS) were obtained by centrifugation of the strains at 13,400 g for 10 min (4°C), followed by filter-sterilization using a sterile filter with a pore size of 0.22 μm. Absorbance (Abs) at 260 nm was measured using an ultraviolet spectrophotometer (Nanodrop ND 2000, Thermo Scientific). The percentage for Abs decrease was calculated as [(A_c_-A_t_)/A_c_×100%)], where A_c_ and A_t_ are absorbance values measured at 0 h and after incubation of a certain time. The media containing the nucleic acid of the same concentration were used as a control (A_c_).

### Nucleic Acid Degradation Assays by Agarose Gel Electrophoresis

The CFS of each strain previously filter-sterilized was used to check its activity for nucleic acid digestion. After incubation for a certain time, the DNA left (without digestion) was analyzed on a 1.2% agarose gel. The reaction yield was obtained by quantitative analysis of the decrease in the intact DNA band (Molecular Imager Gel Doc XR+ imaging system, Image Lab 3.0). The digestion yield was calculated by comparing the intensity of the band with that before digestion.

### Evaluation of Extracellular DNA Degradation Activity Using an Agar Plate Assay

Extracellular DNA degradation activity of *S. aureus* and *E. coli* was examined using a previously described method (Jeffries et al., 1957; Pressler et al., 2019). Briefly, a 10.0 μl aliquot of an overnight culture (washed with PBS buffer (pH 7.2), then resuspended in PBS buffer) was spotted onto nutrient broth agar plates containing 2.0 g/L of DNA and incubated overnight at 37°C. For visualization, plates were flooded with 3.0 ml of perchloric acid solution (50% v/v perchloric acid in distilled water) and kept for 5 min. DNA-digested areas appeared as a clear zone around the colony against an opaque background. The extracellular DNA degradation activity was determined accordingly because there is a direct relation between the diameter of the cleared zone and nuclease activity (Jeffries et al., 1957; Pressler et al., 2019).

### RNA Isolation and Reverse Transcription PCR

Total RNA was extracted from bacteria using RNA preparation pure kit according to the manufacturer's instructions. The RNA samples were diluted with RNase-Free distilled water, and the optical density (OD) values at 260 nm and 280 nm were used to evaluate the RNA concentration and purity. Next, to determine the mRNA expression of each gene, a total of 10.0 μl reverse transcription system was used as per the instructions of the FastKing RT Kit. The generated cDNA was stored at −20°C. RT-qPCR was performed on a PikoReal 96 Real-time PCR System (Thermo Scientific, USA). *16S rRNA* was used for the normalization of mRNA expression, and the relative level of each mRNA was determined using the 2^−ΔΔCt^ method (Landry and Levin, 2014). The PCR conditions were comprised of pre-denaturation at 95°C for 10 min, 40 cycles of denaturation at 95°C for 15 s, annealing at 60°C for 30 s, and extension at 72°C for 30 s. All RT-qPCR experiments were conducted with three duplicate wells, and each gene experiment was repeated three times.

### Statistical Analysis

Data are presented as means ± SEM. The GraphPad PRISM 7.0 software (GraphPad Software Corporation, USA) was used for the analysis of the experimental data. The analysis of variance (ANOVA) was used to estimate the statistical parameters.

## Results

### *Escherichia coli* Uses DNA as the Sole Carbon and/or Nitrogen Source

**Figure 1:**
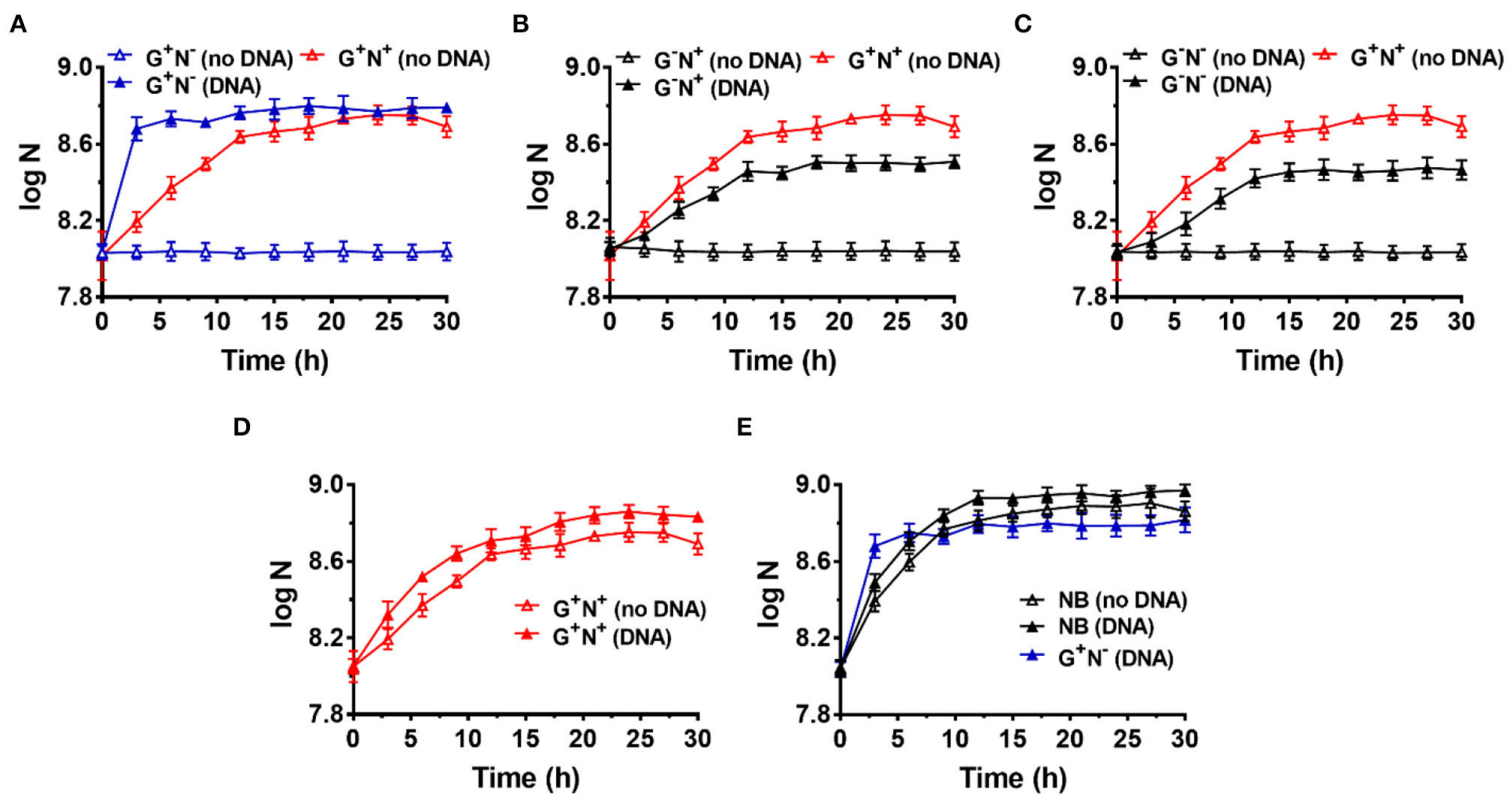
Growth curves for *Escherichia coli* in the presence (DNA) or absence (no DNA) of DNA as the sole carbon and/or nitrogen source in M9 minimal medium. N is the number of bacteria in 1.0 ml. **(A)** Lacking NH_4_Cl (G^+^N^−^). **(B)** Lacking glucose (G^−^N^+^). **(C)** Lacking glucose and NH_4_Cl (G^−^N^−^). **(D)** Complete M9 minimal medium (G^+^N^+^). For **(A–D)**, M9 medium (G^+^N^+^) was used as the positive control (red solid triangles). **(E)** Growth of *E. coli* in nutrient broth (NB) with DNA added as an organic nutrient. For **(E)**, the result is shown for comparison of M9 medium lacking NH_4_Cl (G^+^N^−^) with DNA added as an organic nutrient. The concentrations of DNA and glucose are 0.8 g/L and 4.0 g/L, respectively. Growth of *E. coli* was carried out at 37°C for 30 h. OD_600_ values were measured after certain time intervals. Assays were carried out in triplicate, and average values were used (mean and standard deviation are shown).

To avoid incorporation or entrainment of other carbon (C) or nitrogen (N) sources, we chose the M9 medium (containing 22.0 mM glucose, 18.6 mM NH_4_Cl, 22.0 mM KH_2_PO_4_, 0.10 mM CaCl_2_, 47 mM Na_2_HPO_4_, 8.5 mM NaCl, and 2.0 mM MgSO_4_) as the basal medium, using glucose as the sole C and NH_4_Cl as the sole N source. Glucose is the only organic nutrient in the M9 medium. As shown in Figure 1A (red blank triangles), *E. coli* grew well in M9 within the first 12 h (as the logarithmic phase). Very interestingly, when salmon sperm DNA (100–250 bp long) was used to replace NH_4_Cl, *E. coli* grew much more quickly and the logarithmic phase was as short as about 3 h (blue solid triangles, Figure 1A). In the stationary phase, the OD value was even higher than that in the complete medium of M9. Certainly, when neither DNA nor NH_4_Cl was used, no growth of *E. coli* was observed because no N source was present in the medium (blue blank triangles). This effect depends to some extent on the DNA concentration, and 0.2–1.0 g/L DNA shows a better effect (Supplementary Figure 2A).

In the case that glucose was replaced by DNA (G^−^N^+^, Figure 1B) or both glucose and NH_4_Cl were replaced by DNA (G^−^N^−^, Figure 1C), *E. coli* did not grow so quickly compared with that DNA replacing NH_4_Cl (G^+^N^−^). In addition, the OD values were lower than the positive control (G^+^N^+^, M9 containing glucose and NH_4_Cl) in the stationary phase. Interestingly, even when both NH_4_Cl and DNA were present as the N source, the quick growth as shown in Figure 1A was not observed, and the OD value became a little bit higher than that for M9 (Figure 1D). More interestingly, even for nutrient broth (NB), which is very rich in various nutrients, the growth speed was slower for the first 3 h as compared with the combination of DNA and glucose (see black blank triangles in Figure 1E). Obviously, as compared with NB, the M9 medium contains very poor nutrition. Surprisingly, when both DNA and NB were present, the growth speed was also not so quick in the first 3 h (black triangles in Figure 1E). These results show that the extremely quick growth of *E. coli* only occurs in the case that DNA was used as the sole N source (or nutrient-poor medium) in the presence of glucose.

Notably, even in the case that DNA was the only organic compound, *E. coli* grew well, although both the growth speed and the final concentration were not as good as that in the M9 medium (comparing G^−^N^−^ (DNA) with G^+^N^+^ (no DNA) in Figure 1C). This result demonstrates that DNA can be used as the sole carbon and sole nitrogen source for *E. coli* growth. Amazingly, *E. coli* can grow in the presence of only DNA and inorganic salts. Another exciting result was that even in NB, the addition of DNA could improve both the growth speed and the bacterium density of *E. coli* (comparing black solid triangles and blank triangles in Figure 1E), indicating that DNA can be used as a nutrient (probably being mainly used as the nitrogen source even in the case of rich nutrition). In addition, similar results were obtained when the inoculum was replaced by the following culture, in which *E. coli* was cultured in a medium with DNA as the sole nitrogen source but not complete M9 (Supplementary Figure 3).

### DNA Is Comparable to Glutamic Acid as the Nitrogen Source for *E. coli* Growth

**Figure 2:**
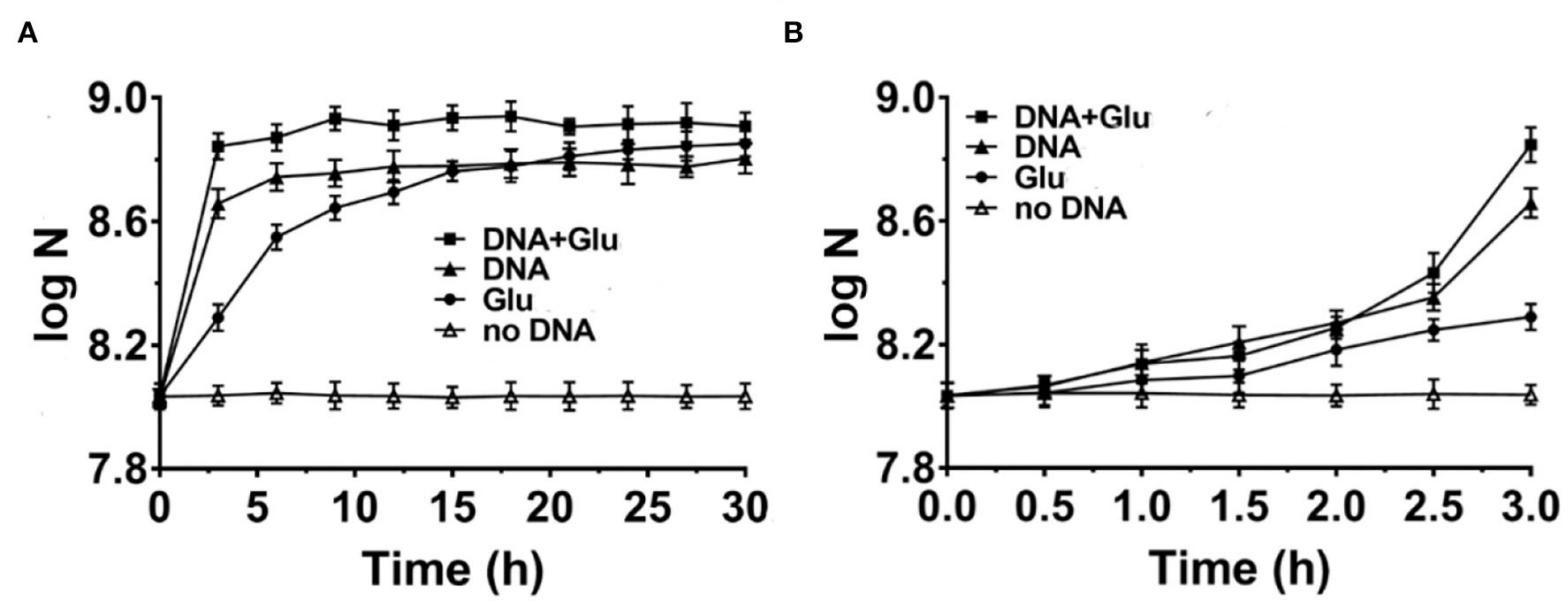
Comparison of DNA with glutamic acid as the nitrogen source for *Escherichia coli* growth in M9 media **(A)**. The enlarged part of the first 3 h is shown in **(B)**. N is the number of bacteria in 1.0 ml. Solid triangles: 0.8 g/L DNA; Solid circles: 1.43 g/L glutamic acid (Glu); Solid squares: both DNA and glutamic acid are present; Blank triangles: no nitrogen source is present. Glucose of 4.0 g/L is used as the carbon source.

It is well known that amino acids are considered the best nitrogen source for bacteria among simple organic compounds. Here, we chose glutamic acid for comparison because it is one of the key compounds for the metabolism of protein, nucleic acids, and glucose. For a more fair comparison, we use 0.8 g/L of DNA (containing about 17% nitrogen on average) or 1.43 g/L of glutamic acid (containing 9.5% nitrogen) with the same concentration of nitrogen (~0.136 g/L). As shown in Figure 2A, when glutamic acid was used to replace DNA, the growth speed (0.30 × 10^8^ CFU/ml/h) for the first 3 h was not as fast as the case for DNA (1.27 × 10^8^ CFU/ml/h) (comparing triangles and circles). Interestingly, when both glutamic acid and DNA were present, the growth speed became much faster (2.0 × 10^8^ CFU/ml/h, see squares in Figure 2A). Similar results were obtained when 0.625–5.4 g/L of glutamic acid was used (Supplementary Figure 2B). Notably, this is different from the cases in that DNA was present together with other nitrogen sources such as NH_4_Cl or components in NB, in which the speed became slower (Figures 1B,C,E). These results show again that DNA can be used as an excellent nitrogen source for *E. coli* at least in some special cases.

As shown in Figure 2B, *E. coli* grew slowly in the first 2 h, indicating that the gene expression of proteins using DNA and/or glutamic acid as a nitrogen source was not ready. Obviously, the difference in growth speed was biggest during the 30 min from 2.5 to 3.0 h of culture. The corresponding average growth speeds for glutamic acid, DNA, and both glutamic acid and DNA were determined to be 0.46 × 10^8^, 4.96 × 10^8^, and 8.80 × 10^8^ CFU/ml/h, respectively (data not shown). This result shows that there is a synergistic effect between DNA and glutamic acid. In addition, it also indicates that *E. coli* can use DNA as an N source very efficiently once *E. coli* prepares well corresponding proteins to digest and utilize DNA. The consumption of DNA and glutamic acid was also estimated. At the stationary phase for DNA as the sole nitrogen source (Figure 2A), about 6.0 × 10^8^ CFU/ml *E. coli* was obtained. Suppose that the volume of an *E. coli* cell is 0.5 μm^3^ and its dry weight is about 1.5 × 10^−13^ g, the dry weight of 6.0 × 10^8^ CFU/ml *E. coli* is equivalent to 0.09 g/L. Accordingly, considering the efficiency of anabolism, it can be estimated that 5–20% of organic nutrition (0.8 g/L DNA and 4.0 g/L glucose) was consumed.

### *Escherichia coli* Can “Eat” DNA Directly and Grow Quickly Using DNA as the Nitrogen Source

How does *E. coli* use DNA in the presence of glucose and grow so quickly? Two possible paths can be proposed. One is that *E. coli* secretes nuclease to digest DNA to oligonucleotides, nucleotides, or nucleosides around the cells, and then these relatively small molecules are ingested. The other one is that *E. coli* ingests long DNA molecules directly without fragmenting them, followed by digestion and utilization in the cells. The latter hypothesis can explain better the quick growth of *E. coli*. To figure out which speculation is correct, we analyzed the change in DNA amount in the medium at various time intervals.

**Figure 3:**
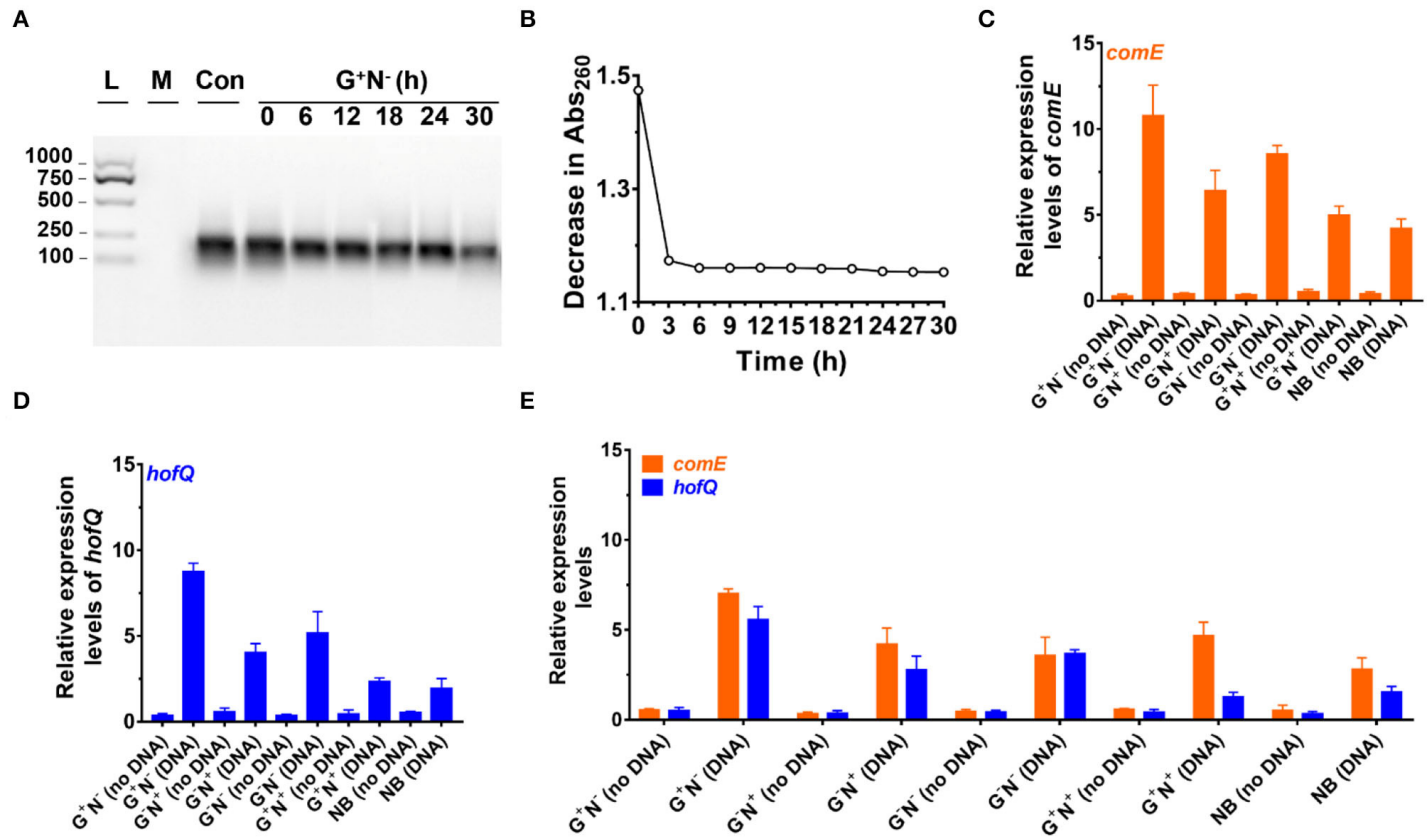
Analysis of DNA uptake to *Escherichia coli*. **(A)** Analysis of DNA size change by electrophoresis analysis (agarose) at various time intervals of culture in the media using DNA as the sole nitrogen source (G^+^N^−^). L: 100–1,000 bp ladder; M: growth media only, Con: control (medium containing DNA without *E. coli*). **(B)** Decrease in absorbance at 260 nm at various time intervals of culture (G^+^N^−^). For **(A)** and **(B)**, the supernatant after centrifugation was used. **(C)** Expression of *comE* (binding protein for extracellular DNA uptake) in *E. coli*. **(D)** Expression of *hofQ* (a protein for extracellular DNA uptake) in *E. coli*. For **(C)** and **(D)**, the *E. coli* after being cultured for 3 h was used. **(E)** Expression of *comE* and *hofQ* genes after 24 h of the culture in various media. *16S rRNA* was used for the normalization of gene expression; the relative level was determined using the 2^−ΔΔCt^ method (Landry and Levin, 2014).

Figure 3A shows the result of electrophoresis analysis of DNA in the culture media (G^+^N^−^, glucose as C source, and DNA as N source) after removing *E. coli* cultured for a certain time. It can be seen that the amount of DNA (100–250 bp) decreased to some extent during culture, but the DNA length did not change. For the first 6 h, the intensity of the DNA band decreased by approximately 20%, and almost no obvious decrease could be observed after that. Considering that the error for quantitative analysis by electrophoresis is large, we further checked the absorbance (Abs) change in the supernatant at 260 nm with the culturing time after removing the *E. coli* by centrifugation (Figure 3B). Interestingly, the Abs decreases with a similar trend during culture as that of the *E. coli* growth curve (Figure 1A), demonstrating that *E. coli* utilized DNA to grow. Again, Abs did not change much after 6 h of culture, indicating that the utilization stopped or reached the equilibrium between intake and release of molecules with Abs at 260 nm. A decrease in DNA was also observed for other culture media (G^−^N^−^, G^−^N^+^, G^+^N^+^, and NB), and the DNA was not digested completely in all cases (Supplementary Figure 4).

We further quantitatively analyzed the expressed mRNA of two proteins (ComE and HofQ) related to DNA uptake by reverse transcription-quantitative real-time PCR (RT-qPCR). Protein ComE (coding by the *comE* gene) is in charge of DNA binding, and protein HofQ (coding by the *hofQ* gene) ingests long dsDNA directly into *E. coli* (Goodgal, 1982; Palchevskiy and Finkel, 2006). As shown in Figures 3C,D, the transcription level for both proteins became much higher in the presence of DNA (cultured for 3 h). In the absence of DNA, even for the NB medium, the transcription level was also much lower. Interestingly, these two proteins were expressed in a similar order in the presence of DNA: G^+^N^−^ > G^−^N^−^ > G^−^N^+^ > G^+^N^+^ ≈ NB. Obviously, the expression of *comE* and *hofQ* was at a higher level when DNA was used as the sole nitrogen source. Surprisingly, their expression was also upregulated even when another nitrogen source (NB or NH_4_Cl) was present, indicating that DNA can be ingested once a relatively high concentration of DNA (e.g., 0.8 g/L) is present. It should be noted that DNA accounts for about 0.5% weight of *E. coli* cells (5.0 g/L).

After 24 h of culture (Figure 3E), even when the growth stopped for G^+^N^−^ (DNA), a high level of expression of *comE* and *hofQ* was kept, although it is a little bit lower than that at 3 h (comparing the data for G^+^N^−^ (DNA) in Figure 3E with those in Figures 3C,D). For all the other media containing DNA, the gene expression of *comE* and *hofQ* was also kept at a relatively high level (Figure 3E). These results indicate that the presence of a high concentration of DNA can stimulate the expression of genes in *E. coli* for DNA uptake. It should be noted that these two proteins only “eat” relatively long dsDNA. Accordingly, it can be concluded that *E. coli* can detect the exogenous DNA and be able to “eat” it as food for growth.

**Figure 4:**
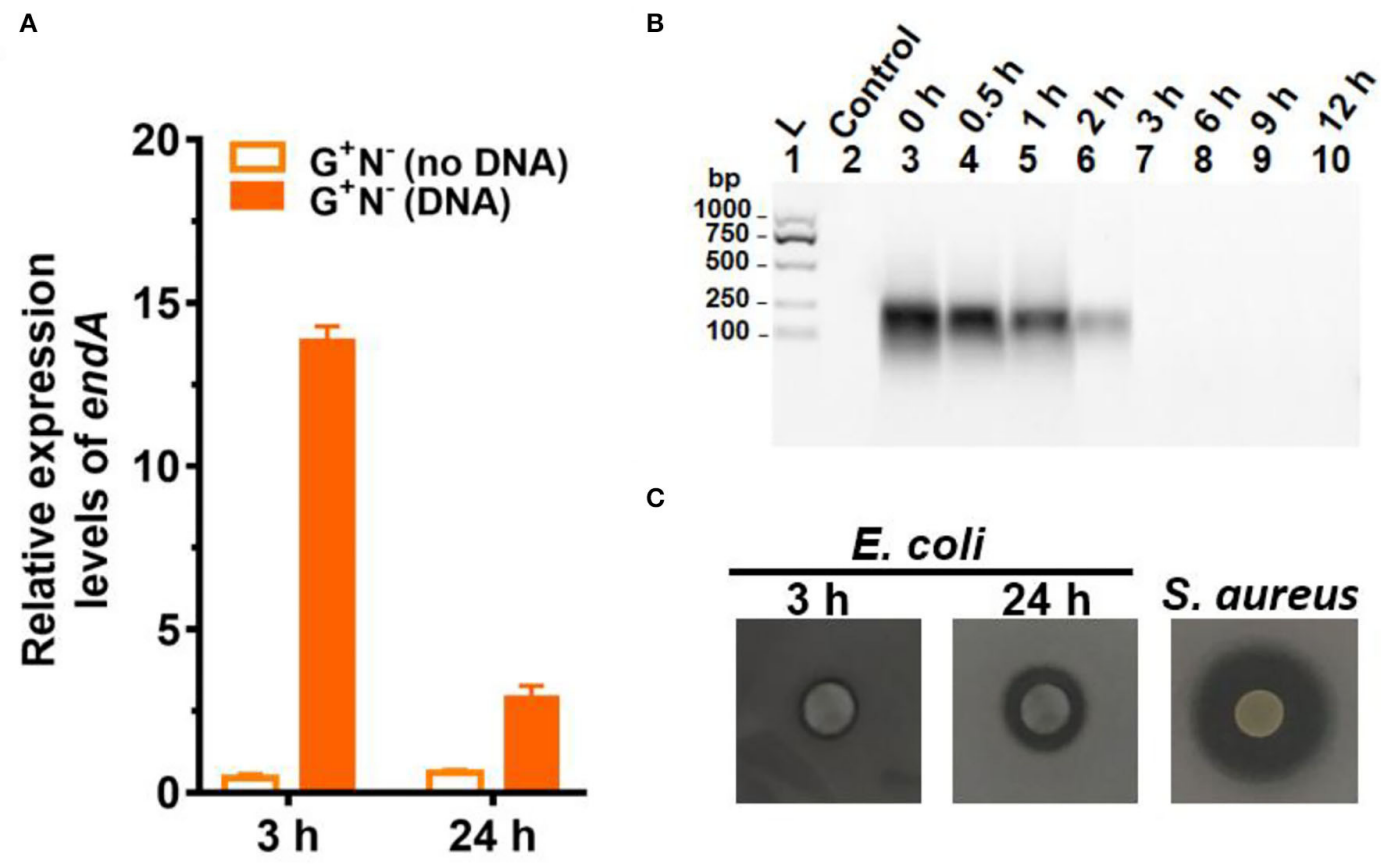
Gene expression and digestion activity of DNase (EndA) from *Escherichia coli*. **(A)** Gene expression of deoxyribonuclease EndA of *E. coli* in the presence or absence of DNA in the medium (G^+^N^−^). *16S rRNA* was used for normalization, and the relative level was determined using the 2^−ΔΔCt^ method (Landry and Levin, 2014). **(B)** The digestion of DNA (0.8 g/L) by DNase (0.01 ng/L) at 37°C for various times. **(C)** Investigation of the extrogenous DNase activity of *E. coli* on agar nutrient broth containing 0.2% (w/v) filter-sterilized DNA. The pictures were taken after incubation at 37°C for 3 h or 24 h. The clear region (black color region here) appearing around the colony reflects the areas where DNA in the agar was digested. *S. aureus* (ATCC6538) for 24 h was used as the positive control.

To check whether *E. coli* secretes deoxyribonuclease (DNase) to digest DNA, the expression of the *endA* gene (to produce EndA protein, an endodeoxyribonulclease) was investigated. EndA has been proposed to be related to utilizing DNA as nutrition (Heun et al., 2012). By gene analysis, we found that the *endA* gene had a high degree of sequence identity with a known DNase of *P. fluorescens* extracellular deoxyribonuclease (VVP09331.1). The deduced amino sequence reveals that this DNase has a potential signal peptide (SignalP 5.0 software, score 0.998), demonstrating that it can be secreted outside the *E. coli* cell (Cherny and Sauer, 2019). As shown in Figure 4A, when DNA was present in the medium, *endA* was expressed at a much higher level, especially after 3 h of culture. After 24 h, however, the expression level decreased greatly. Certainly, in the absence of DNA in the medium, *endA* was only expressed at a background level, which was lower than that of *16S rRNA* (as the base for comparison). These results show that the presence of exogenous DNA did improve the expression level of *endA* to digest DNA (see also Supplementary Figure 5 for the results in other media). However, a new question has arisen about whether the DNA is digested completely outside of the *E. coli*, or in its periplasm. We further tried to clarify this as follows.

It is well known that a tiny amount of DNase can destroy long DNA efficiently because even if 1% digestion occurs, most of the 100 bp DNA should be destroyed to shorter ones. We checked this by adding only 0.01 ng/L of DNase I to the medium containing DNA but without *E. coli* (Figure 4B). As expected, no DNA could be observed after 3 h, showing that all DNA was digested to shorter ones. Obviously, the digestion here was much faster as compared with those shown in Figure 3A, indicating that EndA may be mainly present in the periplasm but not completely outside the cell. This was further proved by checking the ability of *E. coli* to secrete DNase (EndA) by another approach, in which *E. coli* were spotted onto nutrient broth agar containing 0.2% (2.0 g/L) filter-sterilized DNA and kept for 3 or 24 h (Figure 4C). For the *E. coli* cultured for only 3 h, almost no DNase activity was observed; even after 24 h of culture, the DNase activity was much less compared with the positive control of *S. aureus*. Interestingly, when the cells of *E. coli* (G^+^N^−^ (DNA), 3 h) were disrupted, and the liquid of cell disruption was used for DNA digestion, DNA was digested completely even within 1 h (data not shown). More interestingly, even for the control without DNA in the medium, the liquid of cell disruption also showed some DNase activity, indicating that DNase may be present to a certain level in the periplasm whether DNA is present in the medium or not. Certainly, when the cell-free supernatant (DNA is present in the medium or not during culture) was used, almost no digestion was observed even after 30 h of incubation (data not shown).

### Deoxyribonucleotides and Deoxyribonucleosides Are Used as the Excellent Nitrogen Source for *E. coli* Growth

**Figure 5:**
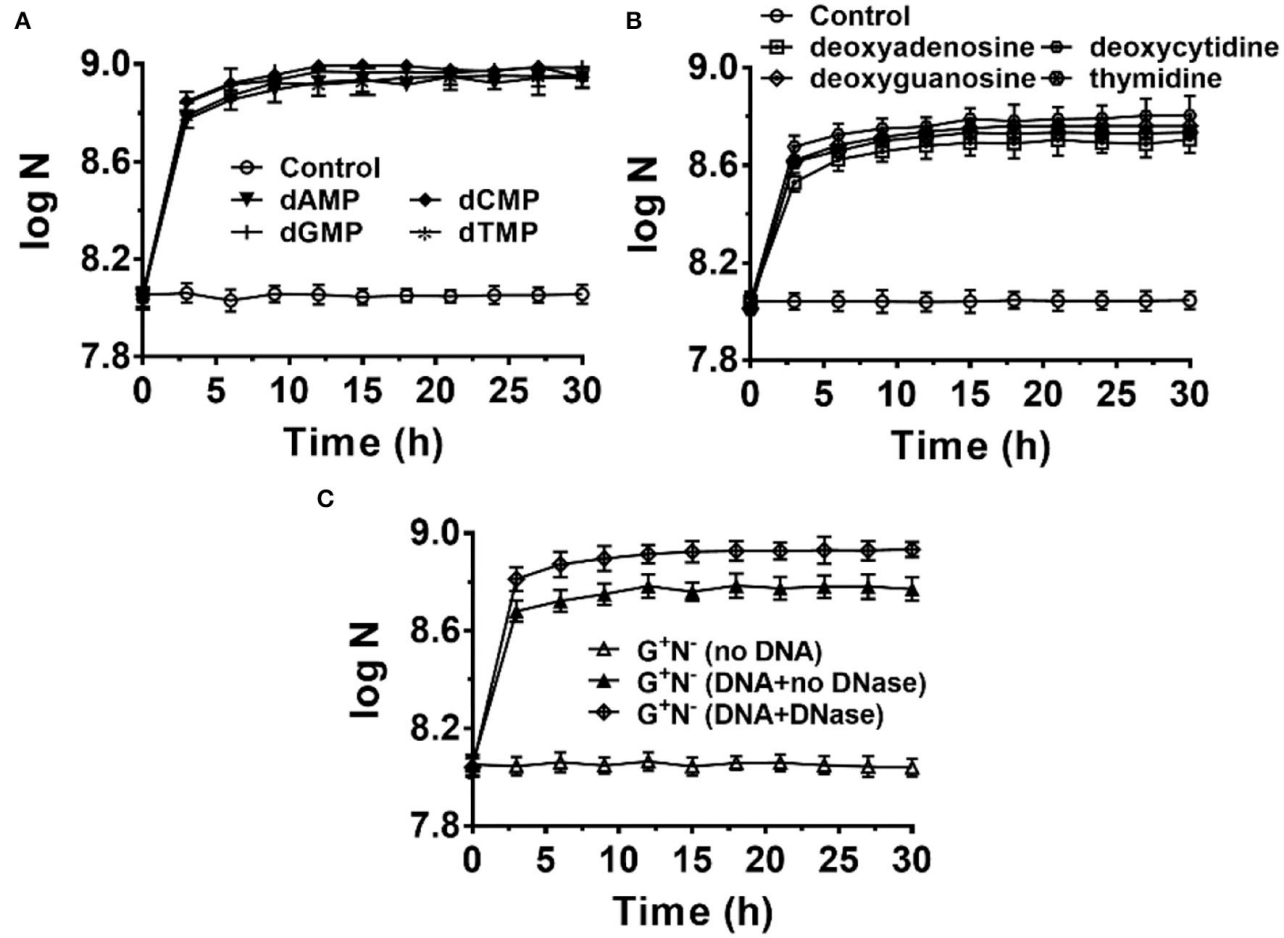
*Escherichia coli* can use deoxyribonucleotides **(A)** and deoxyribonucleosides **(B)** as the sole nitrogen source and 4.0 g/L of glucose as the carbon source for growth. N is the number of bacteria in 1.0 ml. The concentration for each deoxyribonucleotide or deoxyribonucleoside was 1.0 g/L. **(C)** Effect of adding DNase I (0.01 ng/L) to the G^+^N^−^ medium containing DNA on *E. coli* growth.

It is easy to imagine that DNA has to be hydrolyzed into small molecules (monomers and even smaller ones) for synthesizing the macromolecules required for *E. coli* growth. To check whether *E. coli* can ingest directly deoxyribonucleotides (dNMPs) and deoxyribonucleosides, we used dNMPs and deoxyribonucleosides to replace DNA in the medium of G^+^N^−^. As shown in Figure 5A, *E. coli* could grow well using dNMPs in an M9 medium without other nitrogen sources. Similar to the case using DNA as the sole nitrogen source, *E. coli* grew quickly in the first 3 h. No obvious differences were observed for dAMP, dCMP, dGMP, and dTMP. In the case that deoxyribonucleosides (without phosphate) were used, *E. coli* also grew quickly in the first 3 h. However, the final concentrations of *E. coli* were about 40% lower. For *E. coli* growth, *E. coli* can use the phosphate in dNMPs as a P source, but for deoxyribonucleosides media, *E. coli* can only use K_2_HPO_4_ as the P source. Again, no obvious differences were observed for four deoxyribonucleosides (dA, dG, dC, and dT). Similar results were also obtained for other media (G^+^N^−^, G^−^N^−^, G^+^N^+^, NB, Supplementary Figures 6 and 7).

When 0.01 ng/L of DNase I was added to the culture medium of G^+^N^−^ (DNA), both the growth speed and the final concentration of cultured *E. coli* increased to some extent (Figure 5C). This result indicates again that *E. coli* can ingest both big dsDNA molecules (>100 bp) and smaller ones such as dNMPs and deoxyribonucleosides. Similar results were obtained for other media (G^+^N^−^, G^−^N^−^, G^+^N^+^, NB, Supplementary Figure 8). As described previously, in summary, *E. coli* prefers to ingest dsDNA directly and digest it in its periplasm. In contrast, *E. coli* can also ingest and utilize directly the fragments and monomers of DNA, which are present due to other factors. These results indicate again that DNA and its digested products of small molecules are all delicious food for *E. coli*.

### *Bifidobacterium bifidum* (Gram-Positive Bacteria) Can Also Use DNA as the Sole Carbon and/or Nitrogen Source

**Figure 6:**
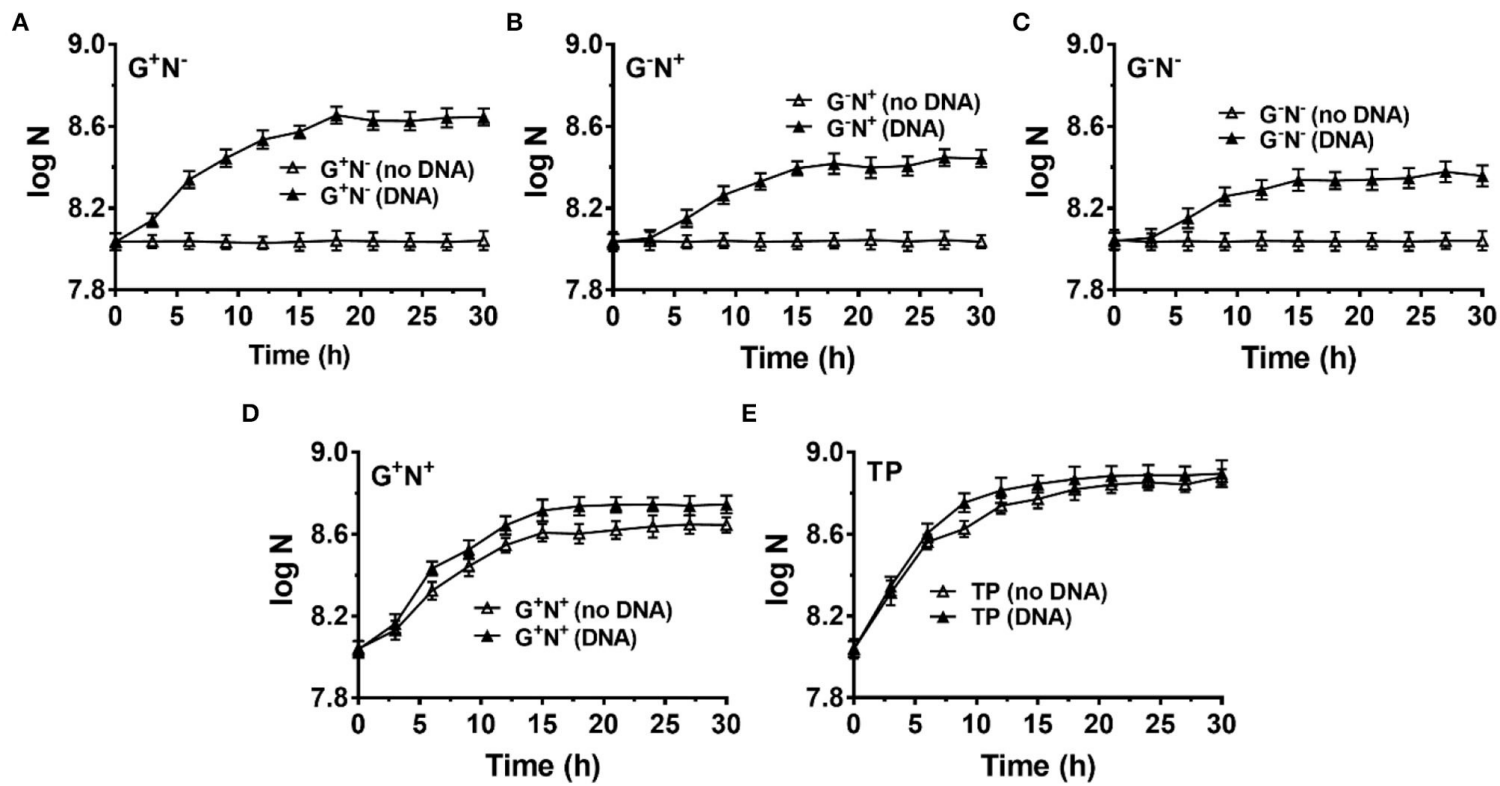
Growth of *Bifidobacterium bifidum* using DNA as the sole carbon and/or nitrogen source. N is the number of bacteria in 1.0 ml. **(A–C)** M9 minimal medium lack of NH_4_Cl (G^+^N^−^), glucose (G^−^N^+^), both glucose and NH_4_Cl (G^−^N^−^), respectively. **(D)** DNA was added into the full M9 minimal medium (G^+^N^+^). **(E)** DNA was added into TPY broth. The concentration of DNA was 0.5 g/L. Media without DNA were used as controls. *B. bifidum* was cultured at 37°C for 30 h under anaerobic conditions. Assays were carried out in triplicate and OD_600_ values were measured.

Finally, we checked whether *B. bifidum* (a gram-positive bacterium) can use DNA as a nutrient (Figure 6). M9 (G^+^N^+^) was also used as the base medium, and NH_4_Cl and/or glucose were removed in some cases (G^+^N^−^, G^−^N^+^, and G^−^N^−^). For all cases, *B. bifidum* grew to a stationary stage after 15–18 h (Figures 6A–C). Interestingly, the final concentration of *B. bifidum* in the case where DNA was used as the sole nitrogen source (G^+^N^−^) was higher than other two cases (G^−^N^+^, G^−^N^−^). In G^+^N^−^ medium, although *B. bifidum* grew faster in the first several hours to some extent compared with G^−^N^+^ and G^−^N^−^ media, the very quick growth (in the case of *E. coli*) was not observed. This result indicates that the direct ingestion of dsDNA may only occur for *E. coli*, which is a kind of gram-negative bacteria with the periplasmic space. When DNA was additionally added to M9 (G^+^N^+^) or TYP media (medium), no obvious synergistic effect of glucose as carbon and DNA as a nitrogen source was observed, indicating again that *E. coli* and *B. bifidum* use DNA differently. In spite of the above results, we can conclude that *B. bifidum* can use DNA for growth, especially in the absence of other nitrogen sources.

## Discussion

Our results clearly show that *E. coli* can ingest dsDNA directly and utilize it as a nutrient, especially as an excellent nitrogen source, which is even comparable to amino acids such as glutamic acid (Figures 1–3). Considering that DNA usually exists in the form of dsDNA either inside or outside a cell (released from organisms), it is more efficient for *E. coli* to “eat” dsDNA to the periplasm and digest DNA there for further utilization. It is less efficient than *E. coli* secreting DNase to the culture medium (outside the cell to the environment) to digest DNA to monomers before ingestion. In addition, secretion of DNase to the environment is not profitable because *E. coli* usually live together with many other bacteria. The digested DNA monomers and fragments are easily snatched by *E. coli*'s competitors. In contrast, the DNase concentration will become much lower if secreted to the culture medium.

Not like proteins or RNA, dsDNA has a very simple secondary structure (duplex) so bacteria can easily evolve proteins to ingest dsDNA directly. Although *E. coli* can also ingest and utilize directly dNMPs or deoxyribonucleosides as the nitrogen source (Figure 5), it does not mean *E. coli* use this approach (digest dsDNA to small molecules and ingest) as the main one for DNA utilization. However, it demonstrates that DNA is a good nutrient and *E. coli* may use any kind of DNA and its derivatives. It can be speculated that DNA and its derivatives are preferred to be utilized even when their concentration is much lower than that in the medium we used.

Although many reports claim that bacteria use their own extracellular nucleases to degrade DNA (Croft et al., 1968; Akrigg and Mandelstam, 1978; Focareta and Manning, 1987; Pinchuk et al., 2008; Mulcahy et al., 2010; Gödeke et al., 2011; Seper et al., 2011; Heun et al., 2012; Korczynska et al., 2012; Liechti and Goldberg, 2013; Jaskólska et al., 2018; Cherny and Sauer, 2019), it is not clear where these nucleases work outside the cell or in the periplasm. For example, *V. cholerae* has been shown to secrete two nucleases (exonuclease Xds and endonuclease Dns) to break down extracellular DNA into nucleotides as a nutrient (Focareta and Manning, 1987; Seper et al., 2011). *Bacillus subtilis* was reported to release DNase to degrade and utilize foreign DNA when it lacks essential nutrients (Akrigg and Mandelstam, 1978). In this study, we pointed out that nuclease EndA is used as the major nucleases to digest extracellular DNA in the periplasm. EndA (a 235-aa protein) contains an N-terminal signal sequence that is predicted to be located in the periplasm, which is also confirmed experimentally (Cordonnier and Bernardi, 1965; Heun et al., 2012). We believe that for most gram-negative bacteria, their periplasm is the main location for DNA digestion. The weak DNase activity in the media may be caused by the release of DNase from dead bacteria or leak from their outer membrane.

Earlier studies reported the abundance of extracellular DNA in both terrestrial and aquatic environments (Dell'Anno et al., 1998; Finkel and Kolter, 2001; Trulzsch et al., 2001; Rittmann et al., 2005; Vlassov et al., 2007), where bacteria may use the DNA as a nutrient. Actually, besides those from food, there are two major sources of DNA in the mammalian gut lumen. One is eukaryotic DNA shed from the mucosal epithelium, and the other pool is the DNA from the indigenous microflora. The amount of eukaryotic DNA has been estimated as ~ 5 mg/day in the stomach, 200–500 mg/day in the small intestine, 20–50 mg/day in the colon, and ~6–15 mg/day in the lumen (Croft et al., 1968; Croft and Cotton, 1973). In some special cases, e.g., acute episodes of diarrhea, eukaryotic DNA can reach 1–10 g/day in the small intestines of humans (Banwell et al., 1970, 1971). From the aspect of bacteria, they should not miss these nutrient-rich materials (especially rich in nitrogen) as food. On average, the content of nitrogen in nucleic acids is higher than that in amino acids or proteins.

This study has confirmed that salmon sperm DNA can be used as nitrogen and carbon sources by *E. coli*, one of the intestinal bacteria. Especially in the case that no other nitrogen source was present, *E. coli* can utilize dsDNA very quickly in the presence of glucose (Figure 1). Here, very simple media are used because we want to make the results clear without the distribution of other organic nutrients. The possibility of *E. coli* utilizing contaminated molecules as a nitrogen source is extremely low because similar results were obtained when artificially synthesized DNA was used (Supplementary Figure 9). The size of DNA seems a very important factor in its utilization. We believe that, even when *E. coli* lives in a nutrient-rich medium, DNA, as well as RNA, can be utilized efficiently. Notably, for most bacteria, DNA and RNA account for 10–20% of their dry weight. The utilization of exogenous nucleic acids to synthesize the DNA and RNA in the bacteria should be energetically favorable. In addition, nitrogen-rich nutrients are usually insufficient compared with carbon sources. Accordingly, we believe that the utilization of DNA, as well as RNA by intestinal flora, is closely related to human health. The nucleic acids in the food may be also a good nutrient for us.

**Figure 7:**
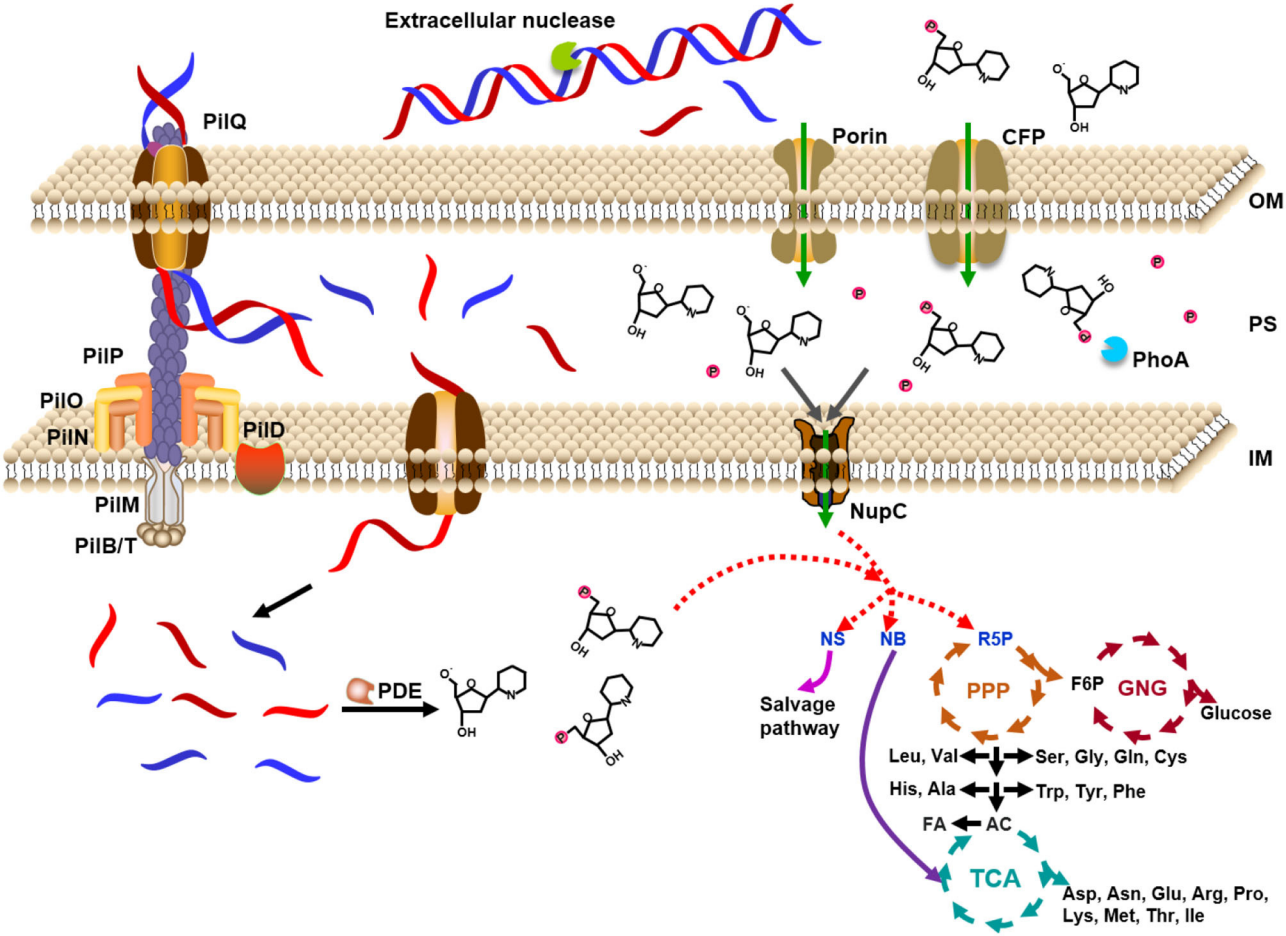
Model for utilization of extracellular DNA as a nutrient by *Escherichia coli*. DNA is caught by proteins on the outer membrane and ingested to the periplasm, where DNase digests DNA into small molecules. The expressed DNase is secreted from the cytoplasm to the periplasm. Small DNA molecules (~7 nt) and monomers are ingested into the cytoplasm by some specific proteins on the inner membrane. After digested to deoxyribonucleosides and even bases and deoxyribose, these small molecules enter the cycles of TCA (tricarboxylic acid cycle), PPP (pentose phosphate pathway), and GNG (gluconeogenesis). The small molecules can also be ingested from the environment directly to the cytoplasm. PDE: *E. coli* phosphodiesterase which hydrolyzes deoxyribonucleic acid only in the single-stranded form to yield mono- and dinucleotides; NupC: nucleoside transporter C; CFP: channel-forming protein; NS: nucleoside; NB: nucleobase; R5P: ribose 5-phosphate; F6P: fructose 6-phosphate; AC: acetyl-CoA; FA: fatty acid; OM: outer membrane; PS: periplasm; IM: inner membrane. Proteins of PII series are in charge of uptake of dsDNA.

Based on the above analysis, as shown in Figure 7, we proposed a model for the utilization of extracellular DNA by *E. coli*. DNA passes across the outer membrane into the periplasm through porins (HofQ, responsible for the transport of DNA across the outer membrane) or nucleoside-specific channel-forming protein (van Alphen et al., 1978; Bremer et al., 1990). The DNase (EndA) in the periplasm can quickly digest dsDNA to short DNA fragments (<10 bp) or deoxyribonucleotides and deoxyribonucleosides, followed by intake to the cytoplasm of *E. coli*. Usually, a DNA duplex shorter than 10 bp is easily dissociated to ssDNA form (Lehman et al., 1962). Accordingly, we believe that the short DNA fragments are mainly transformed into the cytoplasm but not further digested in the periplasm. The deoxyribonucleotides and deoxyribonucleosides are mainly formed in the cytoplasm. Then, these small nitrogen-rich molecules are utilized for the synthesis of various molecules for *E. coli* growth by entering the metabolism cycles. Ribose can be used as the carbon source and nucleobases can be used as the nitrogen source. This speculation is supported by the fact that PDE (phosphodiesterase) has no signal peptide. PDE can only hydrolyze ssDNA in the cytoplasm to yield mono- and dideoxyribonucleotides (Lehman, 1960). The relatively high level of expression of some corresponding genes to further hydrolyze dNMPs, deoxyribonucleosides, and nucleobases (*xdhA, preA, pgm* gene) supports the model we proposed (Supplementary Figure 10). As expected, expression is also improved for the genes for salvage nucleotide synthesis (*deoD, apt, pyrG* gene, Supplementary Figure 11) and *de novo* nucleotide synthesis (*purH, pyrF* gene, Supplementary Figure 12).

The direct ingestion of dsDNA to bacteria has also been reported in a proposed model where the intake DNA is recombined with the bacteria genome (Smith et al., 1981; Goodgal, 1982; Kahn and Smith, 1984; Stewart and Carlson, 1986; Dubnau, 1991, 1999; Solomon and Grossman, 1996; Syvanen and Kado, 1998). In our opinion, this kind of system is mainly used for DNA utilization as a nutrient. Only in the case that the intake dsDNA has almost the same sequences as the bacterium genome, recombination can occur. It seems that over a long evolutionary time, the benefits of maintaining a system for horizontal genetic transfer outweigh the costs. Actually, several groups also claim that this dsDNA ingestion system might serve a nutritional purpose by utilizing extracellular DNA (Redfield, 1993; Solomon and Grossman, 1996; Redfield et al., 1997; Burton and Dubnau, 2010; Allemand et al., 2012). Therefore, it is quite reasonable that *E. coli* and other organisms take advantage of this system to efficiently “eat” dsDNA.

## Conclusion

In conclusion, *E. coli* can utilize DNA as a good nutrient by directly “eating” it. In the periplasm, the eaten DNA is digested and the obtained fragments or monomers are transferred into the cytoplasm as nutrients. The ingested DNA can be used to synthesize almost all the biomolecules for *E. coli*'s reproduction. *Bifidobacterium bifidum* can also use DNA as the nitrogen source and carbon source, although the utilization is carried out differently. DNA and its derivatives should be utilized by most organisms as good nutrients. Many scientific new understandings are expected in the area of nucleic acid metabolism and nutrition. A study on the utilization of RNA as a nutrient by bacteria is underway in our lab.

## Data Availability Statement

The original contributions presented in the study are included in the article/Supplementary Material, further inquiries can be directed to the corresponding author/s.

## Author Contributions

XL and LH developed and designed the experiments for the study. LH and YZ performed the experiments. LH, XD, and XL analyzed and interpreted the data. XL, RA, and LH wrote the paper. All authors contributed to the article and approved the submitted version.

## Funding

This study was supported by the Fundamental Research Funds for Co-construction of Universities in Qingdao to XL; Natural Science Foundation of Shandong Province, China ZR2019BC096 to RA; and National Natural Science Foundation of China 31571937 to XL.

## Conflict of Interest

The authors declare that the research was conducted in the absence of any commercial or financial relationships that could be construed as a potential conflict of interest.

## Publisher's Note

All claims expressed in this article are solely those of the authors and do not necessarily represent those of their affiliated organizations, or those of the publisher, the editors and the reviewers. Any product that may be evaluated in this article, or claim that may be made by its manufacturer, is not guaranteed or endorsed by the publisher.
