# Supplementary material for: Escherichia coli Can Eat DNA as an Excellent Nitrogen Source to Grow Quickly
Source: Front Microbiol. 2022 Jun 28;13:894849. doi: 10.3389/fmicb.2022.894849 (PMC9273947; doi:10.3389/fmicb.2022.894849)
Supplement: Supplementary file 1 [file Data_Sheet_1.pdf]

Supplementary Material

Supplementary Table 1. The sequences of primers used in this study

Name	Sequence (5′→3′)	Length (nt)
16S rRNA-fw	CTGGAACTGAGACACGGTCC	20
16S rRNA-rev	GGTGCTTCTTCTGCGGGTAA	20
endA-fw	CAGTCGGTGAGGTGAATG	18
endA-rev	AGAGTGTCAGGTTGTATTGG	20
comE-fw	GGCTGGCAATGGTGATAG	18
comE-rev	GGCAACGATGATGAGGTAT	19
hofQ-fw	CACTACTGTTGATGCTGATAC	21
hofQ-rev	AGAGAATGTTGCCTTCCTG	19
purH-fw	GAACAGGAACTGCGTGAT	18
purH -rev	GCCAATGCCGATAGTCAT	18
pyrF-fw	TGTGTTCTGCTCAGGAAG	18
pyrF-rev	TCTACCGATTGCGTTACC	18
deoD-fw	GGAAGCGGCTGGTATCTA	18
deoD-rev	ATGTCGTTGAAGGTAGTCTG	20
apt-fw	GCAGTCTGACATCACCAT	18
apt-rev	GGCATGAAATCACGGAATT	19
gpt-fw	GACTGATGCCTTCTGAACA	19
gpt-rev	TGGTTGTCGTGATCGTAG	18
hpt-fw	GCGTCGGTGGAGATATTC	18
hpt-rev	GGAGTCAAGATTGCGGAAT	19
upp-fw	TAGCCTGCTGACTTACGA	18
upp-rev	ATACCGACAACGCTGATG	18
xdhA-fw	CCTTAGCCGTGAAGAGTG	18
xdhA-rev	GACAGAACATCCAGACTATAAC	22
preA-fw	TCGGCTGAAGGAAGATTAC	19
preA-rev	GCGGACAGGAGAAGTTAC	18
pgm-fw	GCTTCTATTGGCGGTCTG	18
pgm-rev	ATCTGCTTGCGATGTTCTT	19

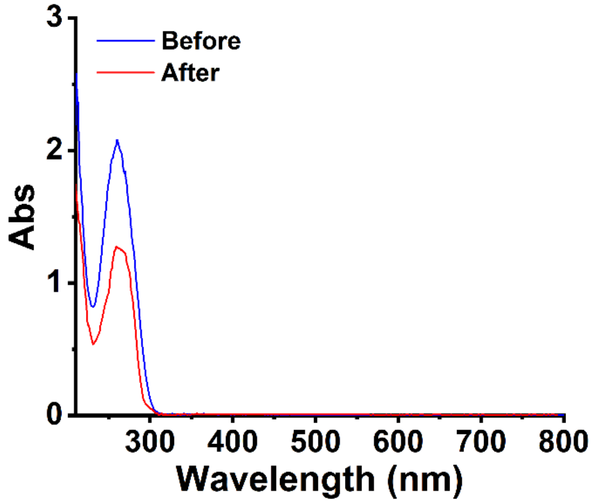

Supplementary Figure 1. **Absorbance spectra of salmon sperm DNA before (blue line) and after (red line) the Proteinase K treatment used in this study.** No side peak was observed, showing that the purity is quite high.

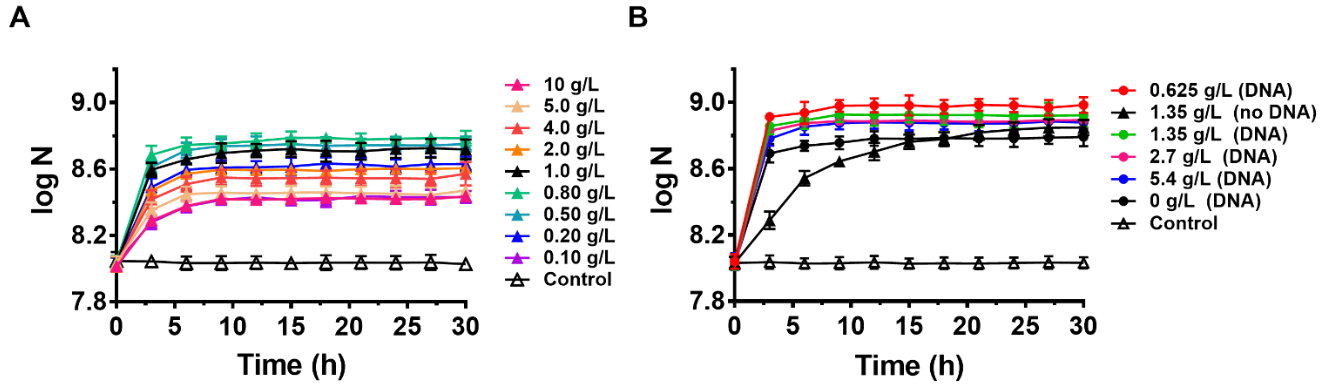

Supplementary Figure 2. **Growth of *E. coli* with DNA as the sole nitrogen source.** (A) Various concentrations of DNA (0.10-10.0 g/L) were used. (B) The concentration of DNA was 0.8 g/L, and glutamic acid concentration was 0.625-5.4 g/L. It seems that *E. coli* prefers to use DNA even in the presence of glutamic acid. The medium without DNA and glutamic acid as control. N is the number of bacteria in 1.0 mL.

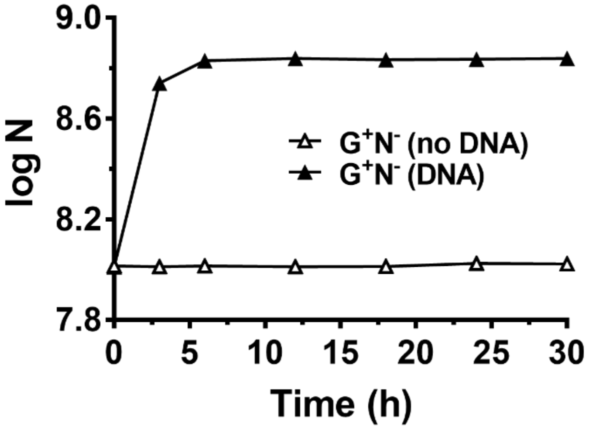

Supplementary Figure 3. **Growth of *E. coli* using the inoculum cultured with DNA as the sole N source.** N is the number of bacteria in 1.0 mL. G^+N^-(DNA): M9 medium using DNA to replace NH_4Cl; G^+N^-(no DNA): M9 medium without NH_4Cl. The inoculum was obtained by culturing *E. coli* in the M9 medium using DNA to replace NH_4Cl.

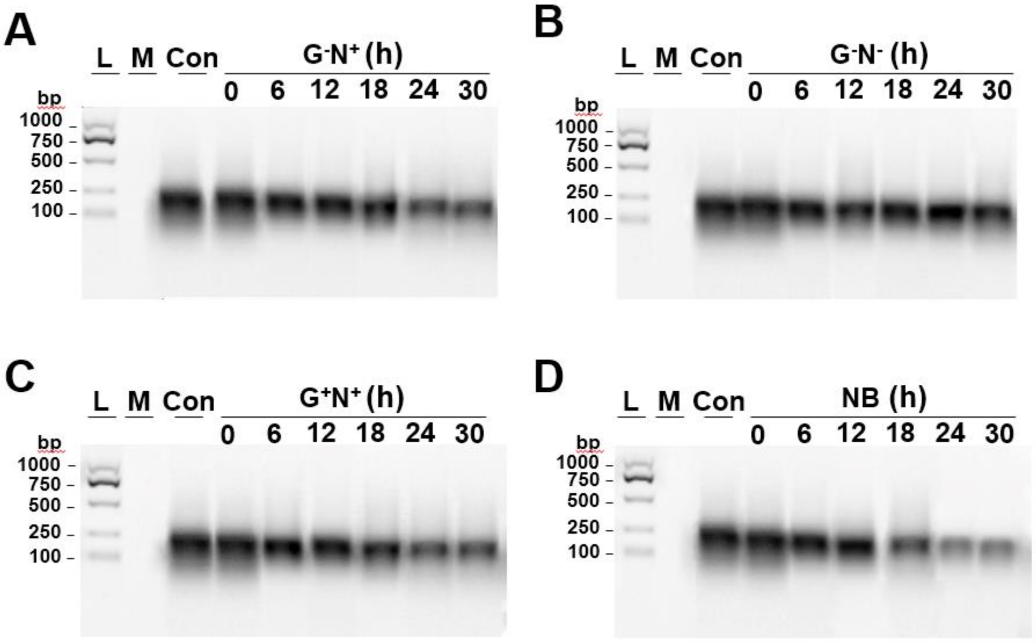

Supplementary Figure 4. **Decrease of DNA concentration in various M9 media after culture of *E. coli*.** (A) Lacking glucose (G^-N^+). (B) Lacking glucose and NH_4Cl (G^-N^-). (C) Complete M9 medium (G^+N^+). (D) Nutrient broth (NB) instead of M9. L: 100-1000 bp ladder; M: growth media without DNA; Con: control (medium containing DNA without *E. coli*).

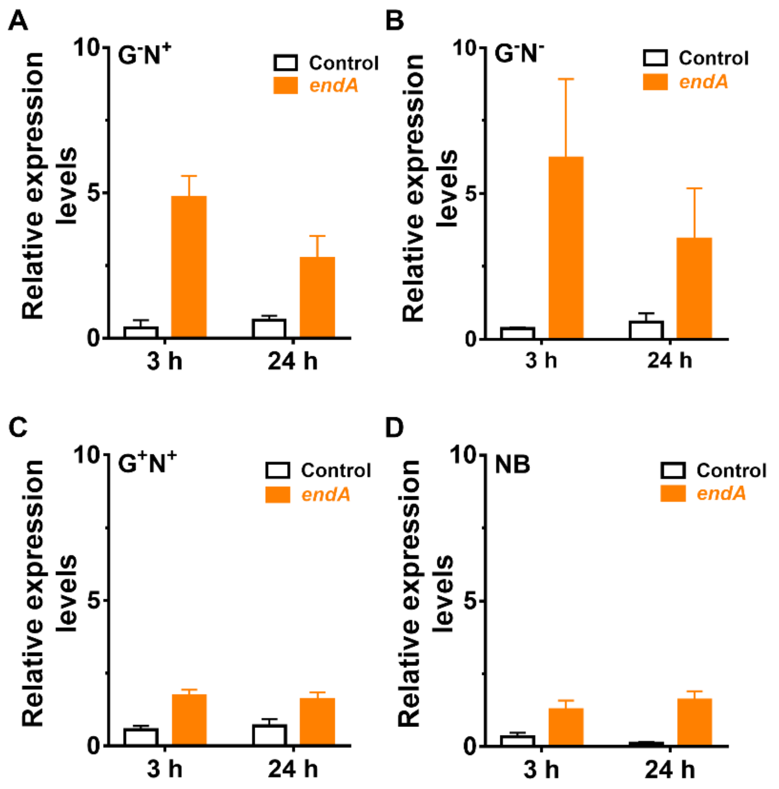

Supplementary Figure 5. **Gene expression of *endA* of *E. coli* after clutured in various M9 media containing DNA for 3 h and 24 h.** (A) Lacking glucose ($G^{-}N^{+}$). (B) Lacking glucose and NH_4Cl ($G^{-}N^{-}$). (C) Complete M9 medium ($G^{+}N^{+}$). (D) Nutrient broth (NB) instead of M9.

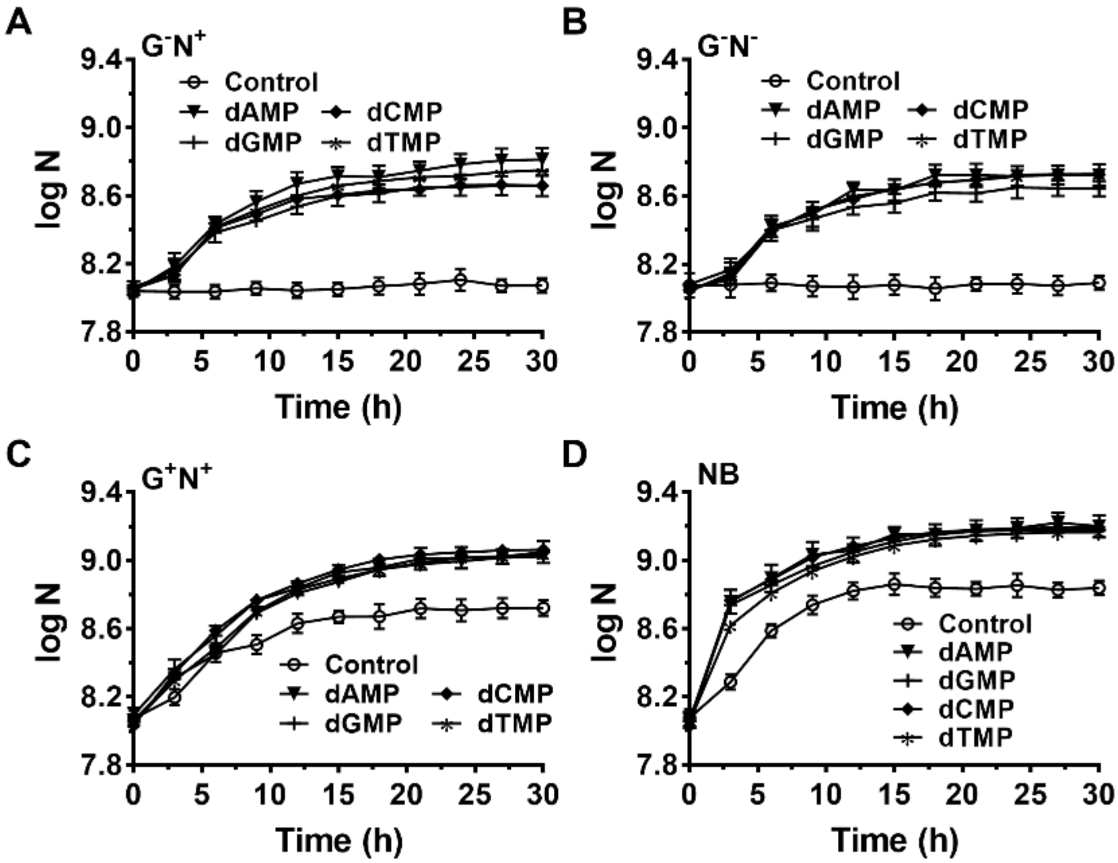

Supplementary Figure 6. Time course of *E. coli* growth in various M9 media containing deoxyribonucleotides (1.0 g/L) as the sole carbon and/or nitrogen source. (A) Lacking glucose (G^-N^+). (B) Lacking glucose and NH_4Cl (G^-N^-). (C) Complete M9 medium (G^+N^+). (D) Nutrient broth (NB) instead of M9. N is the number of bacteria in 1.0 mL.

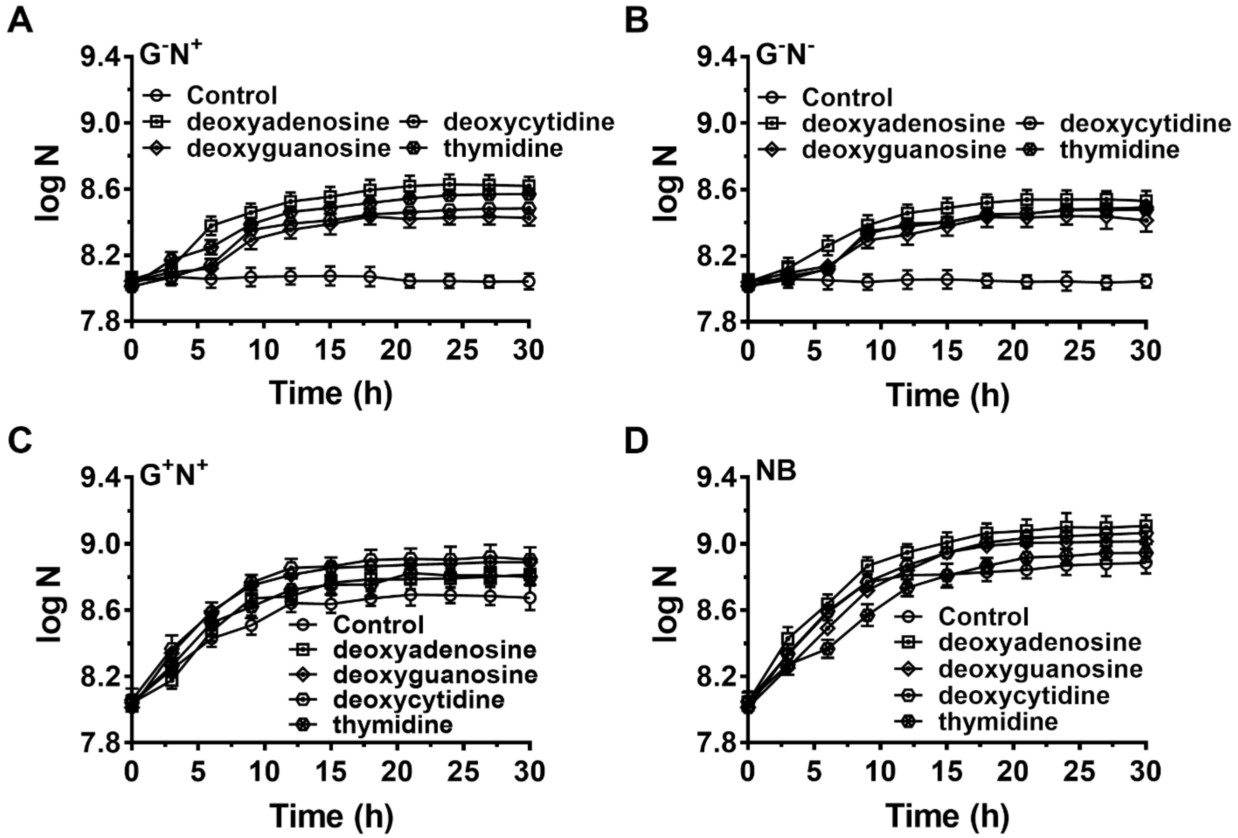

Supplementary Figure 7. **Time course of *E. coli* growth in various M9 media containing deoxyribonucleosides (1.0 g/L) as the sole carbon and/or nitrogen source.** (A) Lacking glucose (G^-N^+). (B) Lacking glucose and NH_4Cl (G^-N^-). (C) Complete M9 medium (G^+N^+). (D) Nutrient broth (NB) instead of M9. N is the number of bacteria in 1.0 mL.

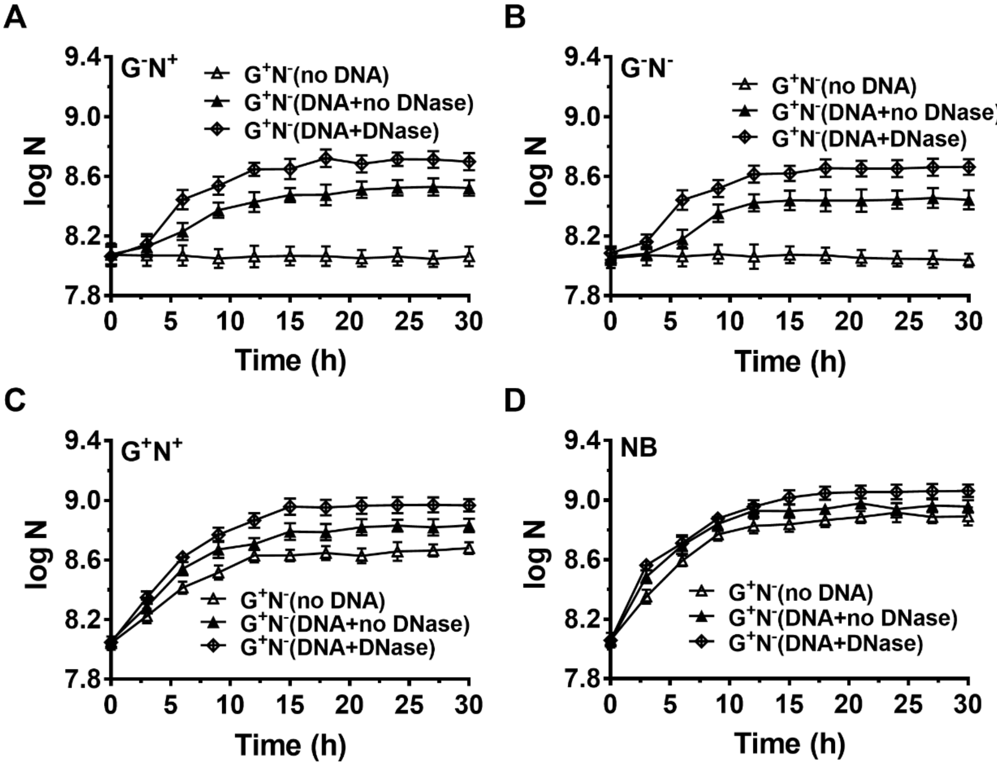

Supplementary Figure 8. **Time course of *E. coli* growth in various M9 media containing DNA in the absence and presence of DNase (0.01 ng/L).** (A) Lacking glucose (G^-N^+). (B) Lacking glucose and NH_4Cl (G^-N^-). (C) Complete M9 medium (G^+N^+). (D) Nutrient broth (NB) instead of M9. N is the number of bacteria in 1.0 mL.

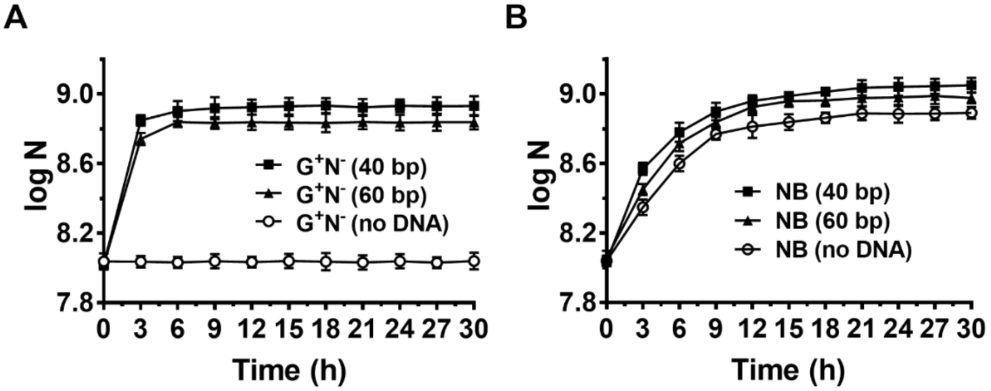

Supplementary Figure 9. **Growth curves for *E. coli* in the presence or absence of DNA (60 bp or 40 bp).** (A) M9 minimal medium without NH_4Cl (G^+N^-) using 40 bp or 60 bp synthesized DNA as the sole nitrogen source. (B) Synthesized DNA (40 or 60 bp) was added in Nutrient broth (NB). The concentration of DNA is 10.0 μM (60 bp DNA is 0.40 g/L and 40 bp DNA is 0.26 g/L). Growth of *E. coli* was carried out at 37°C for 30 h. N is the number of bacteria in 1.0 mL.

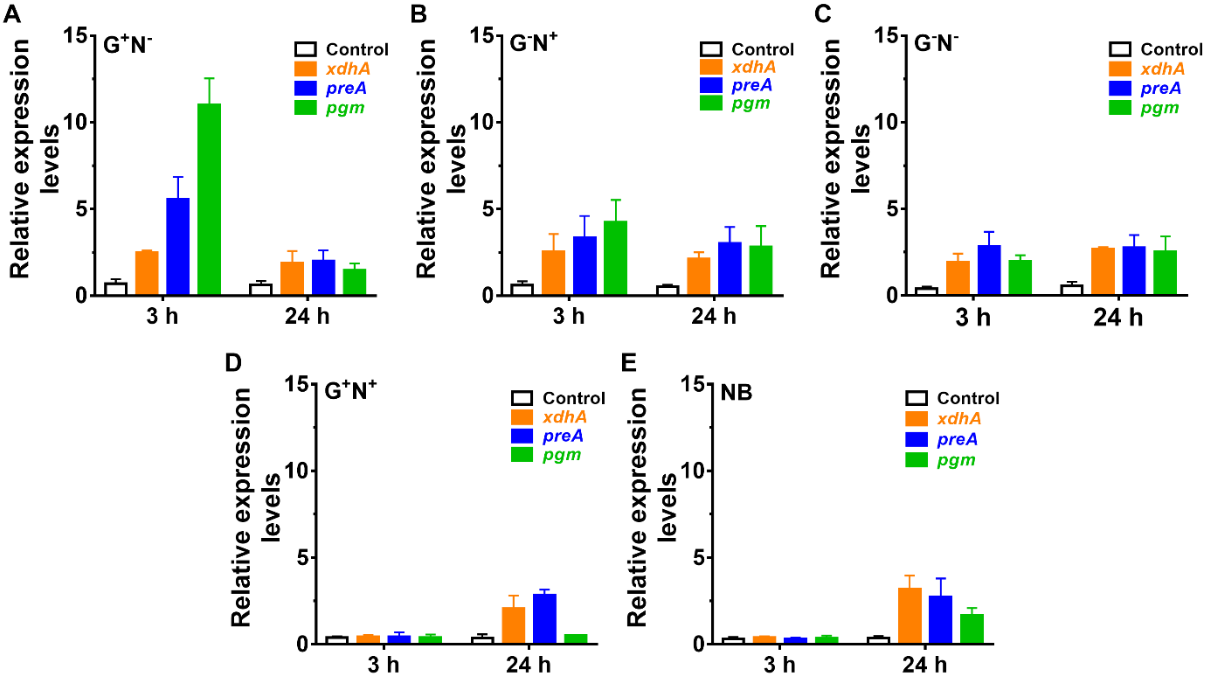

Supplementary Figure 10. Gene expression of nuclotide decomposing enzymes *xdhA*, *preA*, *pgm* of *E. coli* after clutured in various M9 media containing DNA for 3 h and 24 h. (A) Lacking NH_4Cl (G^+N^-). (B) Lacking glucose (G^-N^+). (C) Lacking glucose and NH_4Cl (G^-N^-). (D) Complete M9 medium (G^+N^+). (E) Nutrient broth (NB) instead of M9. The control is gene expression of purine decomposition gene *xdhA* in various M9 media without DNA. The other corresponding controls of *preA* and *pgm* have the similar level of expression, which are not shown for clearness.

In **Supplementary Figure 10**, the expression of *xdhA* (purine decomposition gene), *preA* (pyrimidine decomposition gene) and *pgm* (deoxyribose decomposition gene) which are related to nucleotide decomposition was quantitatively analysed. When DNA was used to replace glucose or NH_4Cl as carbon and nitrogen sources, these genes were all up-regulated (**Supplementary Figures 10A-C**). It indicates that in these media *E. coli* decomposes DNA and uses the produced small molecules as carbon and/or nitrogen source. In the media of M9 and nutrient broth, no nucleotide decomposition genes up-regulation was found after culturing for 3 h (**Supplementary Figures 10D,E**). While after 24 h, *xdhA* and *preA* related to nucleobase decomposition were up-regulated. The possible reason is that the grow speed of *E. coli* was not so quick in the first 3 h (see Figure 1 in the main text), and with the increase of culture time, DNA was ingested so that the nucleobases began to be decomposed and used as the nitrogen source. This result shows that DNA was utilized even in the presence of other nitrogen source, demonstrating that DNA is an excellent nitrogen source. On the other hand, *pgm* (deoxyribose decomposition gene) was not significantly up-regulated in M9 medium (**Supplementary Figure 10D**). The possible reason is that M9 medium contains enough glucose as a direct energy source, and the decomposition of deoxyribose was not stimulated to a high level.

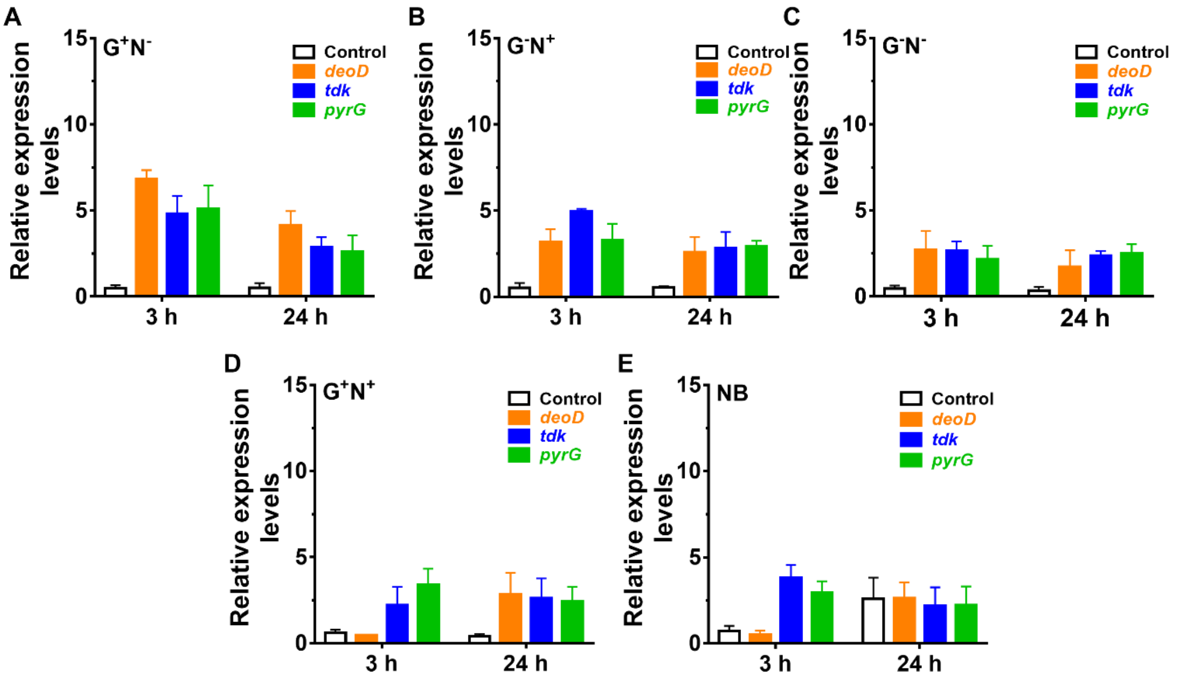

Supplementary Figure 11. Gene expression of salvage nucleotide sythesis enzymes *deoD, tdk, pyrG* of *E. coli* after clutured in various M9 media containing DNA for 3 h and 24 h. (A) Lacking NH_4Cl (G^{+}N^{-}). (B) Lacking glucose (G^{-}N^{+}). (C) Lacking glucose and NH_4Cl (G^{-}N^{-}). (D) Complete M9 medium (G^{+}N^{+}). (E) Nutrient broth (NB) instead of M9. The control is gene expression of deoxyribonucleoside synthesis gene *deoD* in various M9 media without DNA. The other corresponding controls of *tdk* and *pyrG* have the similar level of expression, which are not shown for clearness.

In **Supplementary Figure 11**, the expression of *deoD* (deoxyribonucleoside synthesis gene), *tkd* (deoxynucleotide synthesis gene) and *pyrG* (CTP synthesis gene) which are related to nucleotide salvage synthesis was quantitatively analysed. These genes were up-regulated when DNA was used to replace glucose or NH_4Cl as carbon and nitrogen sources (**Supplementary Figures 11A-C**). In the media lacking carbon and/or nitrogen sources, DNA has to be ingested and decomposed as the nutrient. At the same time, at the stages of either nucleotides or bases being produced, they can be directly used with the salvage pathway for synthesizing dNTPs and NTPs. In M9 and nutrient broth media, *deoD* related to deoxyribonucleoside synthesis was not significantly up-regulated (**Supplementary Figures 11D,E**). The possible reason is that there was not enough deoxyribose to synthesize deoxyribonucleosides. Strangely, genes related to nucleotide salvage synthesis in the control (no DNA) of nutrient broth were also up-regulated, which is hard to explain.

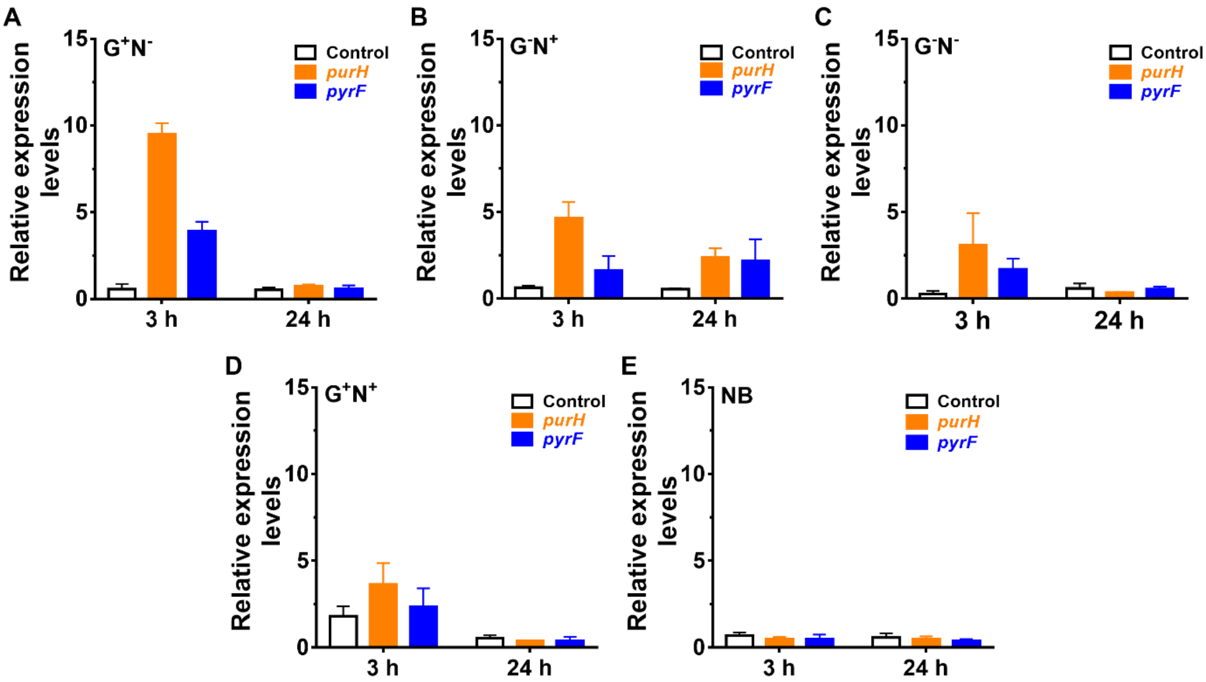

Supplementary Figure 12. **Gene expression of *de novo* nucleotide synthesis enzymes *purH*, *pyrF* of *E. coli* after clutured in various M9 media containing DNA for 3 h and 24 h.** (A) Lacking NH_4Cl (G$^+$N$^-$). (B) Lacking glucose (G$^-$N$^+$). (C) Lacking glucose and NH_4Cl (G$^-$N$^-$). (D) Complete M9 medium (G$^+$N$^+$). (E) Nutrient broth (NB) instead of M9. The control is gene expression of purine de novo synthesis gene *purH* in various M9 media without DNA. The other corresponding control of *purF* have the similar level of expression, which is not shown for clearness.

In **Supplementary Figure 12**, the expression of *purH* (purine *de novo* synthesis gene) and *pyrF* (pyrimidine *de novo* synthesis gene) which are related to nucleotide *de novo* synthesis was quantitatively analysed. These genes were all up-regulated after culturing for 3 h in various M9 media containing DNA (**Supplementary Figures 12A-D**). Especially in the medium lacking NH_4Cl, there is a high level up-regulation of *purH* and *pyrF*, which indicates DNA was utilized as the material for nucleotide synthesis. DNA was decomposed and the produced small molecules were used as the nitrogen source to synthesize amino acids, which is the material for *de novo* RNA synthesis. It should be noted that deoxyribose cannot be oxidized to ribose, but ribose can be reduced to deoxyribose. Because only DNA was present, the *de novo* RNA synthesis pathway has to be active for growth. Interestingly, there was no significant expression in the nutrient broth. The possible reason is that nutrient broth may contain trace amounts of nucleotides, and *E. coli* can use them through the salvage synthesis pathway for growth.
